# Self-Incompatibility in Brassicaceae: Identification and Characterization of *SRK*-Like Sequences Linked to the *S*-Locus in the Tribe Biscutelleae

**DOI:** 10.1534/g3.114.010843

**Published:** 2013-12-23

**Authors:** Jean-Baptiste Leducq, Célia C Gosset, Rita Gries, Kevin Calin, Éric Schmitt, Vincent Castric, Xavier Vekemans

**Affiliations:** * Institut de Biologie Intégrative et des Systèmes, Département de Biologie, PROTEO, Pavillon Charles-Eugène-Marchand, 1030 avenue de la Médecine - Université Laval - Québec (QC) G1V 0A6, Canada; † Institut des Sciences de l’Évolution UMR 5554, CNRS, Université Montpellier 2, Place Eugène Bataillon, C.C. 065 - 34095 Montpellier cedex 05, France; ‡ Laboratoire Génétique et Evolution des Populations Végétales, CNRS UMR 8198, Université Lille1, F-59655 Villeneuve d’Ascq cedex, France

**Keywords:** self-incompatibility, Biscutelleae, SRK, controlled crosses, dominance/recessivity, genetics of sex

## Abstract

Self-incompatibility (SI) is a genetic system that prevents self-fertilization in many Angiosperms. Although plants from the Brassicaceae family present an apparently unique SI system that is ancestral to the family, investigations at the *S*-locus responsible for SI have been mostly limited to two distinct lineages (Brassica and Arabidopsis-Capsella, respectively). Here, we investigated SI in a third deep-branching lineage of Brassicaceae: the tribe Biscutelleae. By coupling sequencing of the SI gene responsible for pollen recognition (*SRK*) with phenotypic analyses based on controlled pollinations, we identified 20 *SRK*-like sequences functionally linked to 13 *S*-haplotypes in 21 individuals of *Biscutella neustriaca* and 220 seedlings. We found two genetic and phylogenetic features of SI in Biscutelleae that depart from patterns observed in the reference Arabidopsis clade: (1) *SRK*-like sequences cluster into two main phylogenetic lineages interspersed within the many *SRK* lineages of Arabidopsis; and (2) some *SRK*-like sequences are transmitted by linked pairs, suggesting local duplication within the *S*-locus. Strikingly, these features also were observed in the Brassica clade but probably evolved independently, as the two main *SRK* clusters in Biscutella are distinct from those in Brassica. In the light of our results and of what has been previously observed in other Brassicaceae, we discuss the ecological and evolutionary implications on SI plant populations of the high diversity and the complex dominance relationships we found at the *S*-locus in Biscutelleae.

Self-incompatibility (SI) is a genetic system that prevents self-fertilization in plants (reviewed in Charlesworth 1987) and is present in approximately 40% of Angiosperm species (reviewed in Igic *et al.* 2008). SI is generally controlled by a single locus (*S*-locus) at which tightly linked genes forming coadapted haplotypic combinations encode pollen and pistil specificities. Pollen is rejected after pollination if its specificity is encoded by the same haplotype as that of the pistil. The determination of pollen phenotype occurs in two distinct flavors in SI systems. In gametophytic systems, the pollen phenotype is defined by its haploid genotype (reviewed in Franklin-Tong and Franklin 2003), whereas in sporophytic systems (SSIs), pollen phenotypes are controlled by the diploid genotype of the paternal plant (reviewed in Hiscock and Tabah 2003).

The molecular mechanism of pollen rejection in SSI has been described only in Brassicaceae and involves the interaction of a cysteine-rich protein (*SCR*) deposited on the pollen surface (Schopfer *et al.* 1999) with a transmembrane receptor kinase (*SRK*) of the stigma (Takasaki *et al.* 2000). Knowledge about the genes involved in SI has allowed comparative investigations of molecular diversity of both genes in different groups of Brassicaceae. Functional alleles of *SRK* have been found in several genera of Brassicaceae, including Brassica (Kusaba *et al.* 1997; Sato *et al.* 2002), Raphanus (Lim *et al.* 2002), Capsella (Guo *et al.* 2009; Paetsch *et al.* 2006), Arabidopsis (Castric and Vekemans 2007; Kusaba *et al.* 2001; Schierup *et al.* 2001), Arabis (Tedder *et al.* 2011), as well as functional alleles of a *SRK* ortholog in Leavenworthia (Busch *et al.* 2008), suggesting that the *SCR/SRK* SSI system is ancestral in this family. *SRK* alleles in these genera share the property of *trans*-specific or even *trans*-generic polymorphisms, as expected due to the strong negative frequency-dependent selection occurring at the *S*-locus (Schierup *et al.* 1998). However, phylogenetic relationships among alleles differ in different groups. SI species of Arabidopsis and Capsella have *SRK* alleles distributed into many lineages, with highly diverged sequences, whereas *SRK* alleles in Brassica and Raphanus, taken together, cluster into only two sequence clades, called classes I and II, evolutionary distinct since they are intermingled with *SRK* lineages from Arabidopsis and Capsella (Figure 1 and Supporting information, Table S1) (Castric and Vekemans 2007; Edh *et al.* 2009; Schierup *et al.* 2001). In the genus Leavenworthia, the *S* alleles clustered into a single clade that is highly divergent from *SRK* alleles from the other genera (Busch *et al.* 2008). This seems to be due to independent evolution of the pollen and pistil genes involved in SI, from different members of the same gene families as *SCR* and *SRK* (Chantha *et al.* 2013).

**Figure 1:**
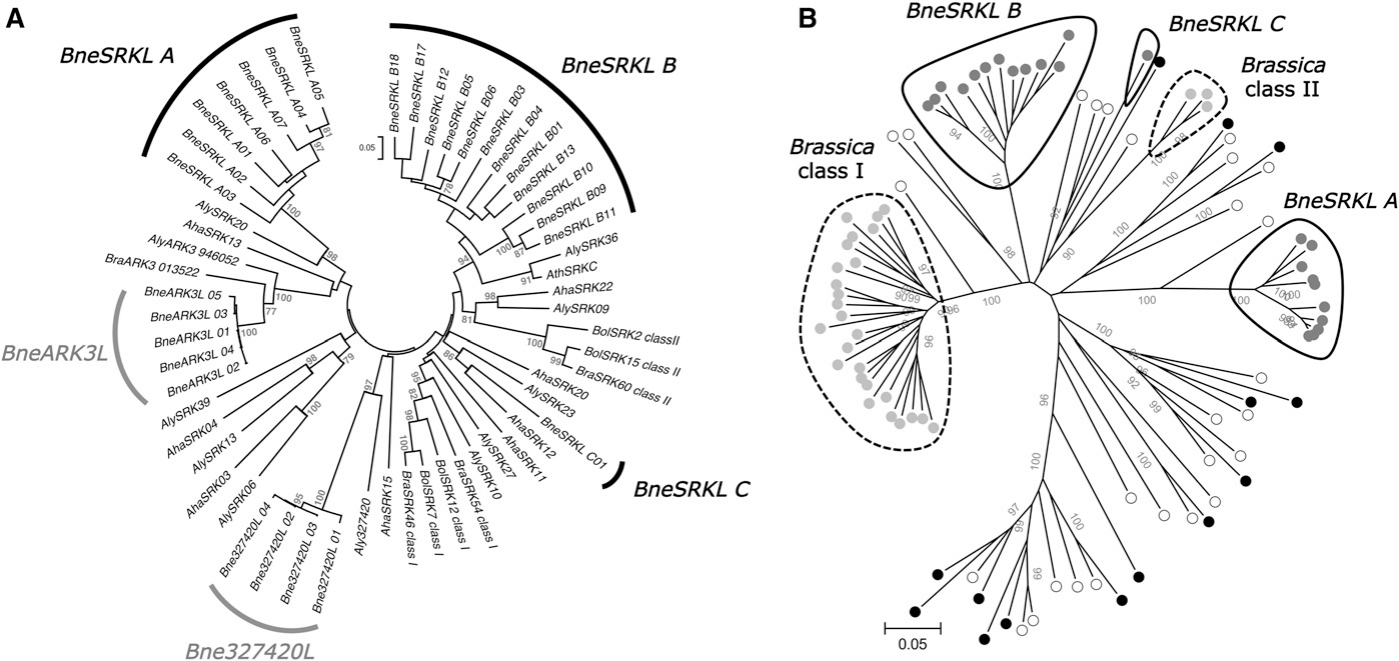
Phylogenetic relationships between the 29 *SRK*-related sequences found in *Biscutella neustriaca* and sequences from other Brassicaceae. The evolutionary trees were built using Neighbor-Joining with a Poisson corrected distance based on amino acid sequences. The proportion of replicate trees in which branches clustered together is indicated close to nodes (1000 bootstrap replicates). (A) The 29 *SRK*-related sequences found in *B. neustriaca* cluster in three monophyletic groups similar in sequence to *SRK* (*BneSRKL_A*, *B*, and *C*) and paralogs *ARK3* and *Aly327420* found in other other Brassicaceae. *Aha*, *Arabidopsis halleri*; *Aly*, *A. lyrata*; *Ath*, *A. thaliana*; *Bol*, *Brassica oleracera*; *Bra*, *B. rapa*; *Bne*, *Biscutella neustriaca*). (B) Phylogenetic relationships among *SRK* and *SRK*-like (*SRKL*) sequences in four species of Brassicaceae: *Capsella grandiflora* (black); *Brassica oleracera* (gray), *Arabidopsis lyrata* (white), *and B. neustriaca* (dark gray). The three clades in Biscutella (continuous line) are distinct to the two clades of Brassica (dotted line). Accession numbers of *SRK*-related sequences are detailed in Table S1.

In SI species of Arabidopsis, only two functional genes, *SRK* and *SCR*, have been found within the *S*-locus (Goubet *et al.* 2012; Guo *et al.* 2011), whereas most Brassica haplotypes have a third gene (*SLG*), which is a paralog of *SRK* lacking its transmembrane and kinase domains (Nishio and Kusaba 2000; Takasaki *et al.* 2000). Although *SLG* is not required for the SI reaction (Nishio and Kusaba 2000), the tightly intermingled phylogeny of *SRK* and *SLG* alleles observed in Brassica suggests frequent gene conversion events between these two genes (Sato *et al.* 2002; Takuno *et al.* 2008).

Another distinctive feature of SSI systems is the occurrence of dominance relationships between *S*-haplotypes in heterozygous genotypes. SI Brassica species have two main dominance classes of *S*-haplotypes, which correspond to the two phylogenetic clusters of haplotypes (Nasrallah *et al.* 1991). In Arabidopsis, dominance relationships appear to be more complex with more than two dominance levels, showing only partial congruence with phylogenetic clustering of alleles (Llaurens *et al.* 2008; Prigoda *et al.* 2005). Dominance relationships are expected to affect *S*-haplotypes frequencies because the negative frequency-dependent selection typical for SI systems (Wright 1939) will be more intense on dominant than on recessive haplotypes (reviewed in Billiard *et al.* 2007), resulting in greater frequencies of recessive haplotypes in natural populations (Schierup *et al.* 1997). This prediction has been validated empirically in *Arabidopsis halleri* (Llaurens *et al.* 2008), *A. lyrata* (Mable *et al.* 2003; Schierup *et al.* 2006), and *Brassica insularis* (Glemin *et al.* 2005).

Although SI is well described and documented in Brassicaceae, there is still little clue about the evolutionary mechanisms responsible for the differences between species just outlined, *e.g.*, regarding the patterns of phylogenetic clustering of alleles, the co-occurrence of a *SRK* paralog at the *S*-locus, and the patterns of dominance relationships. Because most studies focused on two divergent model groups (Brassica-Raphanus and Arabidopsis-Capsella, respectively) we investigates SSI in *Biscutella neustriaca*, a self-incompatible member of the distantly related Biscutelleae tribe (Franzke *et al.* 2011). In a previous study, we defined eight phenotypic incompatibility groups by using a full diallele crossing design between 21 individuals of *B. neustriaca*, which implies that the species has at least eight functional *S*-haplotypes (Leducq *et al.* 2010).

We used molecular approaches to identify *SRK*-like sequences in *B. neustriaca* individuals, together with segregation analyses, controlled pollinations and paternity assignment from open pollinations to relate the sequences to SI phenotypes and to investigate dominance among haplotypes. Two striking features of the *SRK*-like sequences were observed: (1) most haplotypes appear to carry two linked *SRK*-like sequences; and (2) all but one of the *SRK*-like sequences grouped into distinct phylogenetic clusters whose sequences differ from Brassica ones.

## Materials and Methods

### Identification of SRK-like sequences

We used 21 *B. neustriaca* individuals (collection F0) from a collection of plants maintained in the *Conservatoire Botanique National de Bailleul* (France) that we previously studied by controlled pollinations to identify incompatibility groups (Leducq *et al.* 2010). We used these plants to identify putative *SRK* sequences and validate their SI function by investigating association with the incompatibility groups.

DNA was extracted from leaf material as described in Leducq *et al.* (2010). Candidate *SRK* sequences were initially obtained by polymerase chain reaction (PCR) amplification with primers *13SEQ2* and *SLGR* as described in Schierup *et al.* (2001). The PCR mixture (15 µL) contained 20 ng of DNA, 1X buffer (Applied Biosystems, Foster City, CA), 2.5mM MgCl_2_, 400 μM Fermentas dNTP mix (Fermentas Canada, Burlington, ON, Canada), 150 µg/mL BSA, 0.5 mΜ each primer, and 0.05 U/μL Taq polymerase (Amplitaq DNA polymerase; Applied Biosystems). Amplifications were performed on a Mastercycler EpGradient Eppendorf thermocycler with the following conditions: 15 min at 95°, 40 cycles of three steps: (1) 45 sec at 95°, (2) 1 min at 50° and (3) 1 min at 72°, followed by 10 min at 72°. PCR products were purified using NucleoSpin Extract II kit from Macherey-Nagel and ligated and transformed into chemically competent bacteria as described in Castric and Vekemans (2007). At least eight clones per individual were sequenced with the BigDye3.1 sequencing kit (Applied Biosystems) and loaded on a 3130 capillary sequencer. Sequences were edited using MEGA5 (Tamura *et al.* 2011) and validated when identical copies were found in at least two clones.

Validated sequences were checked for similarity with previously described sequences of *SRK* and *SRK*-related genes from other Brassicaceae species using blast searches against the nucleotide database in Genbank, and against the genome of *A. lyrata* at the Phytozome website (http://www.phytozome.net/). In all positive clones sequenced in a first set of 16 individuals (135 clones), sequences from one type (*BneARK3L*) were found in all individuals (60% of all clones). This sequence is closely related to that of *ARK3*, a paralog of *SRK* (also called *Aly8* in *A. lyrata*, and corresponding to NCBI gene ID 946052), which is not involved in pollen-pistil recognition (Charlesworth *et al.* 2003; Kusaba *et al.* 2001). We therefore developed a method to minimize amplification of this gene before the cloning step intended to obtain *SRK* sequences. We identified a restriction site that was present only in this sequence and digested the DNA (purified PCR product) with the *Hin*dIII restriction enzyme (A-AGCTT). The restriction was carried out overnight at 37° in a mixture (20 µL) containing 15 µL of fresh purified PCR product, 0.2 mM Spermidin Sigma, 2 µL of 1X Fermentas R buffer, and 0.05 U/μL *Hin*dIII Fermentas restriction enzyme. Restriction products were mixed with loading dye and run at 110 V on 2% agarose gels in TBE buffer for 45 min. Fragments were visualized by ethidium bromide under ultraviolet light and compared with a 100-bp DNA ladder. Digested PCR products of *BneARK3L* gave two fragments (150 and 450 pb size). Undigested PCR fragments (≈600 pb size) were extracted from the agarose gel, cloned, and sequenced as described previously. Of the 269 positive clones obtained, only 6% contained *BneARK3L*, but 37% contained another sequence type (*Bne327420L*) that is similar to an *A. lyrata* gene with unknown function (Hu *et al.* 2011). We designed specific primers for *Bne327420L* to test whether it segregates at the *S*-locus and it proved to be unlinked (Table S2). The reaction mixture for PCR amplification (15 µL) contained 20 ng of DNA, 1X buffer (Applied Biosystems), 2.5mM MgCl_2_, 200 μM Fermentas dNTP mix, 200 µg/mL bovine serum albumin, 0.5 mm of each primer, and 0.025 U/μL Taq polymerase (Applied Biosystems). Amplifications were performed on Eppendorf thermocycler with the following conditions: 15 min at 95°, 35 cycles of three steps: (1) 40 sec at 95°, (2) 40 sec at 60° and (3) 40 sec at 72°, and finally 10 min at 72°. PCR products were visualized as described previously.

We performed a phylogenetic analysis of all our *B. neustriaca* sequences, together with a sample of *SRK* alleles and *SRK*-related genes from Arabidopsis and Brassica species (Table S1). We used PhyML 3.0 (Guindon *et al.* 2010) on amino acid sequences with the LG model of substitution and 100 bootstraps. We identified several clades of *SRK*-related sequences from Biscutella by characterizing the largest monophyletic groups containing exclusively sequences from *B. neustriaca*. We estimated synonymous (*π*_S_) and nonsynonymous (*π*_N_) nucleotide divergence between sequences identified as belonging to the different clades, using DNAsp v.5 (Librado and Rozas 2009). For putative *SRK* alleles, we checked for signatures of positive selection at the codon level using CODEML from the PAML package (Yang 2007) by comparing the likelihood of the nested models M7 and M8, which differ by the existence in M8 of a category of codon sites under diversifying selection (ω = *dN*/*dS* > 1). Putatively selected codons were determined by the Bayes empirical Bayes inference procedure (Yang *et al.* 2005). Based on annotation of hypervariable regions previously identified in Brassica *SRK* alleles (Kusaba *et al.* 1997), we determined whether putatively selected codons were located within hypervariable regions.

Based on the *SRK*-like sequences, we developed a typing strategy by designing primers with Primer3 (http://primer3.sourceforge.net/) to specifically target each candidate *SRK* sequence identified in collection F0 (Table S2). PCR amplifications were performed as described previously for *Bne327420L*, and individuals were genotyped by absence/presence of a band on agarose gel. Because of the low divergence between some sequences, some nonspecific amplifications occurred (revealed by frequent or systematic amplification in all genotyped individuals, regardless of their incompatibility type). In these cases, PCR products were systematically sequenced, as described previously, using the primers used for the amplifications.

### Segregation of *SRK*-like sequences and association with the incompatibility phenotype

Our second aim was to verify that *SRK*-like sequences are indeed allelic and that they segregate with the SI phenotype. For this we used segregation patterns in F1 offspring to test for associations between the SI phenotype and the occurrence of particular *SRK*-like sequences in the F0 collection. We also tested whether dominance occurs between the *B. neustriaca S*-haplotypes, as we previously inferred (Leducq *et al.* 2010). For this purpose, we used two collections of seedlings obtained from 13 individuals of the F0 collection (Table S3), representing the eight previously identified incompatibility groups (Leducq *et al.* 2010). The first collection of 82 seedlings (F1o) was obtained from open pollinations among seven F0 individuals (four pollen donors and three pollen receptors) and paternities were assigned *a posteriori* with parentage analysis (Leducq *et al.* 2010). This collection included eight groups of verified full sibs. The second collection consisted of five sets of 138 seedlings (F1c) obtained from controlled pollinations among five pairs of compatible F0 individuals. These controlled crosses were performed as described in Leducq *et al.* (2010).

To test whether observed genotype frequencies at the *S*-locus followed Mendelian expectations in the 13 cohorts of F1 individuals, we defined *n* as cohort size (which range from 5 to 50 in collection F1, see Table S3) and randomly generated 100,000 hypothetical cohorts of individuals for each *n* value. Each simulated set of progeny (which we denote as a ‘cohort’) was assumed to be produced by a cross between two diploid individuals A and B with known genotype at the *S*-locus (or binary combinations of four possible *S*-haplotypes *S1*, *S2*, *S3*, and *S4*). We performed the analysis for two cases, depending on the number of different genotypes at the *S*-locus observed in the cohort: (1) individuals A and B both heterozygous (*e.g.*, genotypes *S1S2* and *S3S4*); (2) parent A homozygous (*S1S1*) and parent B heterozygous (*S3S4*; considered only when only one or two genotypes were present in the sibship). Each sibship from a cross could thus include either (1) genotypes *S1S3*, *S1S4*, *S2S3*, and *S2S4* with expected frequency of 0.25 each, or (2) genotypes *S1S3* and *S1S4* with expected frequency of 0.5 each. For each cohort size *n* and each possible genotype, we used the distribution of simulated genotype frequencies to determine whether observed genotype frequencies differed significantly from those expected.

To test for associations between the putative *S*-haplotypes and the SI phenotypes in the offspring, we performed 1472 controlled pollinations between individuals of collection F0, F1o, and F1c. A total of 1229 pollinations were performed between individuals putatively sharing at least one *S*-haplotype, and the others were between individuals not thought to share *S*-haplotypes, to control for pollen viability and stigma receptivity. In collection F0, we had 18 different combinations between 13 putative *S*-haplotypes. We made crosses between different individuals with the same *S*-locus genotype for 21 out of 35 genotypes tested. Controlled pollinations and determination of compatibility were performed as described in Leducq *et al.* (2010).

## Results

### Identification of SRK-like sequences in *B. neustriaca*

Overall, our approach yielded 29 distinct sequences in the F0 individuals (accession numbers KF905295−KF905324; Table S4). These sequences clustered into five clades, defined as the largest monophyletic groups containing exclusively sequences from *B. neustriaca*. Two clades were closer to sequences of *SRK* paralogs than to *SRK* alleles from Arabidopsis or Brassica (Figure 1A). One of these clades includes five sequences (*BneARK3L*) that are similar to the *ARK3* gene sequence in *B. rapa* (gene *Bra 013522*) and *A. lyrata* (*Aly8* or NCBI gene ID *946052*). A second clade of four sequences (*Bne327420L*) is similar to a *SRK* paralog present in the *A. lyrata* genome (gene *327420*) that lack a kinase domain, unlike *ARK3*. Specific PCR primers for *Bne327420L* yielded amplification products in all individuals from collection F0 (data not shown), confirming that these do not correspond to *S*-locus alleles. The 20 remaining sequences were similar to those from *SRK* alleles from several Arabidopsis species, and we will refer to those as *SRK*-like (*SRKL*) sequences (Figure 1A). *B. neustriaca SRKL* sequences formed three distinct clades, which we call classes A, B, and C. The seven class A sequences are similar to *AlySRK20*, a functional *SRK* allele of *A. lyrata* (also called *SRKb* in Kusaba *et al.* 2001). There were 12 class B sequences, which are closest to *AthSRKC*, one of the three alleles still segregating at the *S*-locus in *A. thaliana* (Shimizu *et al.* 2004), and to *AlySRK36*, a functional allele in *A. lyrata* (Bechsgaard *et al.* 2006). The third clade, class C, is represented by a single sequence similar to *AhSRK12*, a functional *A. halleri SRK* allele (Castric and Vekemans 2007). All three clades of *SRK*-like sequences in *B. neustriaca* are distinct from those of Brassica (Figure 1B).

### Segregation of *SRK*-like sequences from F0, F1_C_, and F1_O_ collections

Patterns or presence and absence results for the 20 *SRKL* sequences among the 21 individuals of collection F0 are presented in Table 1. Despite the fact that *B. neustriaca* is diploid (Leducq *et al.* 2013; Tremetsberger *et al.* 2002), we observed up to four *SRKL* sequences per individual, sometimes including A, B, and C classes. However, we observed consistent associations between certain sequences. Hereafter, we denote a putative *SRK* sequence in a simpler form, for example using *A01* as the abbreviation for *BneSRKL_A01*, where A refers to class A. In this notation, we found, for instance, that *A01* is associated with either *A02* or *A03*, and *B01* was always associated with *B13*, *B04* with *C01*, *B10* with *B11*, and *B09* with *B12* (Table 1). Genotyping of the sibships confirmed these associations and provided two more associations (*A06* with *A07* and *B17* with *B18*). These associations suggest linkage disequilibrium between eight pairs of sequences, forming the following haplotypes at a single locus: *A01−A02*, *A01−A03*, *A06−A07*, *B01−B13, B04−C01*, *B10−B11*, *B09−B12*, and *B17−B18* (Table 2). Most of the associations involve sequence pairs from the same class (A or B). However, one haplotype (*B04-C01*) did not. In addition, several sequences identified in the F0 collection, *A04*, *A05*, *B03*, *B05*, and *B06*, were not transmitted in pair. Using information about the eight haplotypes described previously, plus the five distinct haplotypes involving the five sequences just mentioned, allowed us to infer the genotype of each of the 21 F0 individuals (Table 1, Table 2, and Table 3).

**Table 1:** *SRK*-like sequences identified in the F0 collection

Individual	Sequences Identified	Putative Genotypes
1	*B09*, *B10*, *B11**^a^*, *B12*	*B09B12 / B10B11*
2	*B09*, *B10*, *B11**^a^*, *B12*	*B09B12 / B10B11*
3	*B10*, *B11*	*B10B11 / B10B11*^*c*^
4	*A01a*, *A03*, *B10*, *B11*	*A01aA03 / B10B11*
5	*A01b*, *A03*, *B09*, *B12*	*A01bA03 / B09B12*
6	*A01a*, *A02*, *A01a*, *A03**^a^*	*A01aA02 / A01aA03*
7	*A01a*, *A03*, *B06*	*A01aA03 / B06*
8	*A06*, *A07*, *B06*	*A06A07 / B06*
9	*B06*, *B18*	*B06 / B17**^b^**B18*
10	*B01*, *B06*	*B01B13**^b^* */ B06*
11	*B01*, *B13*, *B04*, *C01*	*B01B13 / B04C01*
12	*A05*, *B01*, *B13*	*A05 / B01B13*
13	*A05*, *B04*, *C01*	*A05 / B04C01*
14	*A05*, *B04*, *C01*	*A05 / B04C01*
15	*A05*, *B05*	*A05 / B05*
16	*A05*, *B05*	*A05 / B05*
17	*B04*, *C01*	*B04C01 / B04C01**^c^*
18	*A01a*, *A02*, *B04*, *C01**^a^*	*A01aA02 / B04C01*
19	*B03*, *B06*	*B03 / B06*
20	*B03*, *B05*	*B03 / B05*
21	*A04*, *B01*, *B13*	*A04 / B01B13*

Putative genotypes are deduced from patterns of segregation in the F1c and F1o collections (see Table 3).

a Typed but not sequenced.

b Supposed by association.

c Homozygote genotypes.

**Table 2:** Pattern of *SRK*-like sequences segregation observed in 13 cohorts of seedlings issued from crosses between individuals of collection F0

Seedling Collection	n*^a^*	Parents*^b^*		Seedlings Genotype (Observed Frequency)*^c^*				Deduced Linkage Groups			
								Parent 1		Parent 2	
		1	2	1	2	3	4	1	2	1	2
F1o	7	3	6	*A01A02*	*A01A03*	*–*	*–*	*B10*	*B10*	*A01*	*A01*
				*B10B11*	*B10B11*			*B11*	*B11*	*A02*	*A03*
				(4)	(3)						
	13	3	7	*A01A03*	*B06B10*	*–*	*–*	*B10*	*B10*	*A01*	*B06*
				*B10B11*	*B11*			*B11*	*B11*	*A03*	
				(7)	(6)						
	5	8	4	*A01A03*	*A06A07*	*A01A03*	*–*	*A06*	*B06*	*A01*	*B10*
				*A06A07*	*B10B11*	*B06*		*A07*		*A03*	*B11*
				(2)	(1)	(2)					
	7	8	6	*A01A03*	*A01A02*	*A01A03*	*–*	*A06*	*B06*	*A01*	*A01*
				*A06A07*	*B06*	*B06*		*A07*		*A02*	*A03*
				(1)	(3)	(3)					
	21	9	4	*A01A03*	*B10B11*	*A01A03*	*B06B10*	*B06*	*–**^d^*	*A01*	*B10*
				(4)	(1)	*B06*	*B11*			*A03*	*B11*
						(10)	(6)				
	7	9	6	*A01A02*	*A01A03*	*A01A02*	*–*	*B06*	*–**^d^*	*A01*	*A01*
				(3)	(2)	*B06*				*A02*	*A03*
						(2)					
	6	10	4	*A01A03*	*B01B10*	*A01A03*	*–*	*B01**^d^*	*B06*	*A01*	*B10*
				*B01*	*B11*	*B06*				*A03*	*B11*
				(1)	(4)	(1)					
	16	10	6	*A01A02*	*A01A03*	*A01A02*	*A01A03*	*B01**^d^*	*B06*	*A01*	*A01*
				*B01*	*B01*	*B06*	*B06*			*A02*	*A03*
				(2)	(6)	(6)	(2)				
F1c	14	4	20	*A01A03*	*A01A03*	*B03B10*	*B05B10*	*A01*	*B10*	*B03*	*B05*
				*B03*	*B05*	*B11*	*B11*	*A03*	*B11*		
				(3)	(2)	(4)	(5)				
	30	12	8	*A05A06*	*A05B06*	*A06A07*	*B01B06*	*A05*	*B01*	*A06*	*B06*
				*A07*	(6)	*B01*	(11)			*A07*	
				(7)		(6)					
	30	17	18	*A01A02*	*B04C01*	*–*	*–*	*B04*	*B04*	*A01*	*B04*
				*B04C01*	(17)			*C01*	*C01*	*A02*	*C01*
				(13)							
	50	18	1	*A01A02*	*A01A02*	*B04B09*	*B04B10*	*A01*	*B04*	*B10*	*B09*
				*B09B12*	*B10B11*	*B12C01*	*B11C01*	*A02*	*C01*	*B11*	*B12*
				(11)	(15)	(9)	(15)				
	14	21	8	*A06A07*		*A04A06*		*A04*	*B01**^d^*	*A06*	*B06*
				*B01*	*B01B06*	*A07*	*A04B06*			*A07*	
				(4)	(1)	(5)	(4)				

Collection F1o (eight cohorts) was obtained from open pollinations and seedlings were assigned by paternity analyses (Leducq *et al.* 2010). Collection F1c was obtained by controlled crosses. For each cohort, the following information is shown: number of seedlings, name of F0 parental individuals, *SRK*-like sequences found in parents, associations of *SRK*-like sequences found in progeny with frequency of association and deduced *SRK*-like linkage groups in parents

a Cohort size.

b F0 collection; see Table 2 for genotypes.

c F1 collection.

d B13, B17, and B18 could not be typed in progeny.

**Table 3:** Occurrence of putative *S*-haplotypes identified in F0 collection (sequencing)

*S*-Haplotype	Sequence		Incompatibility Group	Haplotype Co-occurrence in F0	Cohorts With the Haplotype*^a^*
	1	2			
*S01*	*B10*	*B11*	I	5	7
*S02*	*A01*	*A03*	II	4	9
*S03*	*B06*	*–*	III	5	9
*S04*	*B01*	*B13^b^*	IV	4	2*^b^*
*S05*	*A05*	*–*	V	5	1
*S06*	*B04*	*C01*	VI	6	2
*S07*	*A01*	*A02*	VII	2	4
*S08*	*B03*	*–*	VIII	2	1
*S09*	*A06*	*A07*	–	1	4
*S10*	*B05*	*–*	–	3	1
*S11*	*B09*	*B12*	–	3	1
*S12*	*B17^b^*	*B18^b^*	–	1	0*^b^*
*S13*	*A04*	*–*	–	1	1

Incompatibility groups previously identified in F0 collection by Leducq *et al.* (2010) are indicated with associated *S*-haplotypes.

a Number of cohorts where segregation could be tested; see Table 2 and Table S4.

b B13, B17, and B18 were not typed in the progeny.

These results indicate that *B. neustriaca* has two *SRKL* genes in most of its *S*-locus haplotypes, and that most individuals we analyzed are heterozygotes. In two individuals (3 and 17) we found only one haplotype (haplotypes *B10−B11* and *B04−C01*, respectively), so we hypothesize that these individuals could either be homozygotes for those haplotypes or heterozygotes with another haplotype, so far undetected (Table 1 and Table 2).

We tested the 13 putative *S*-haplotypes inferred from *SRKL* sequences in sibships F1c and F1o. In eight of ten sibships the genotype frequencies followed Mendelian expectations from crossing two individuals heterozygous at the *S*-locus (case 1), but two F1o sibships had significant deviations frequencies (one for one genotype, and one for two). In three other sibships from crossing one heterozygous individual and individuals 3 or 17, for which only a single haplotype was identified, genotype frequencies of 0.5 were found (case 2) and the alternative of more than two progeny genotypes was rejected (Table S3). This confirms that these two individuals are homozygotes for their respective haplotypes. Thus, all *S*-locus genotypes were inferred in the F0 collection.

### Associations between SRK-like sequences and SI phenotypes

As shown in Figure 3, the eight incompatibility groups (groups I−VIII) identified from *S* phenotypes in the F0 collection (Leducq *et al.* 2010) correspond to groups of individuals sharing at least one *SRKL* sequence (putative *SRK* allele) and we will now refer to these as *S*-haplotypes *S01* to *S08* (Table 3).

**Figure 2:**
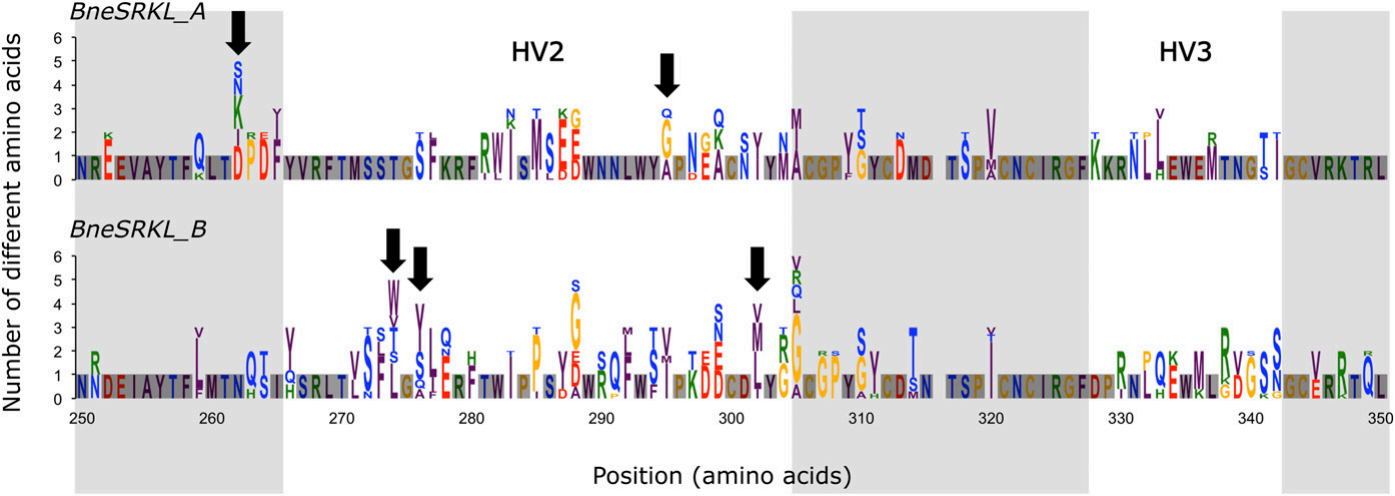
Amino acid variation in two *SRK*-related sequence groups found in *Biscutella neustriaca*. Height of bars is proportional to the number of different amino-acids at each position within a group. Relative frequency of each amino acid (symbolized by its letter) is proportional to its height in the bar. Black arrows indicate positions under positive selection in groups A and B (see Table 4) that fall into or close to hypervariable regions known to be involved in *SRK-SCR* protein recognition (HV2 and HV3 indicated by white frames). The alignment (100 amino-acids positions) was based on eight *BneSRKL-A* and 11 *BneSRKL-B* sequences in MEGA5.

**Figure 3:**
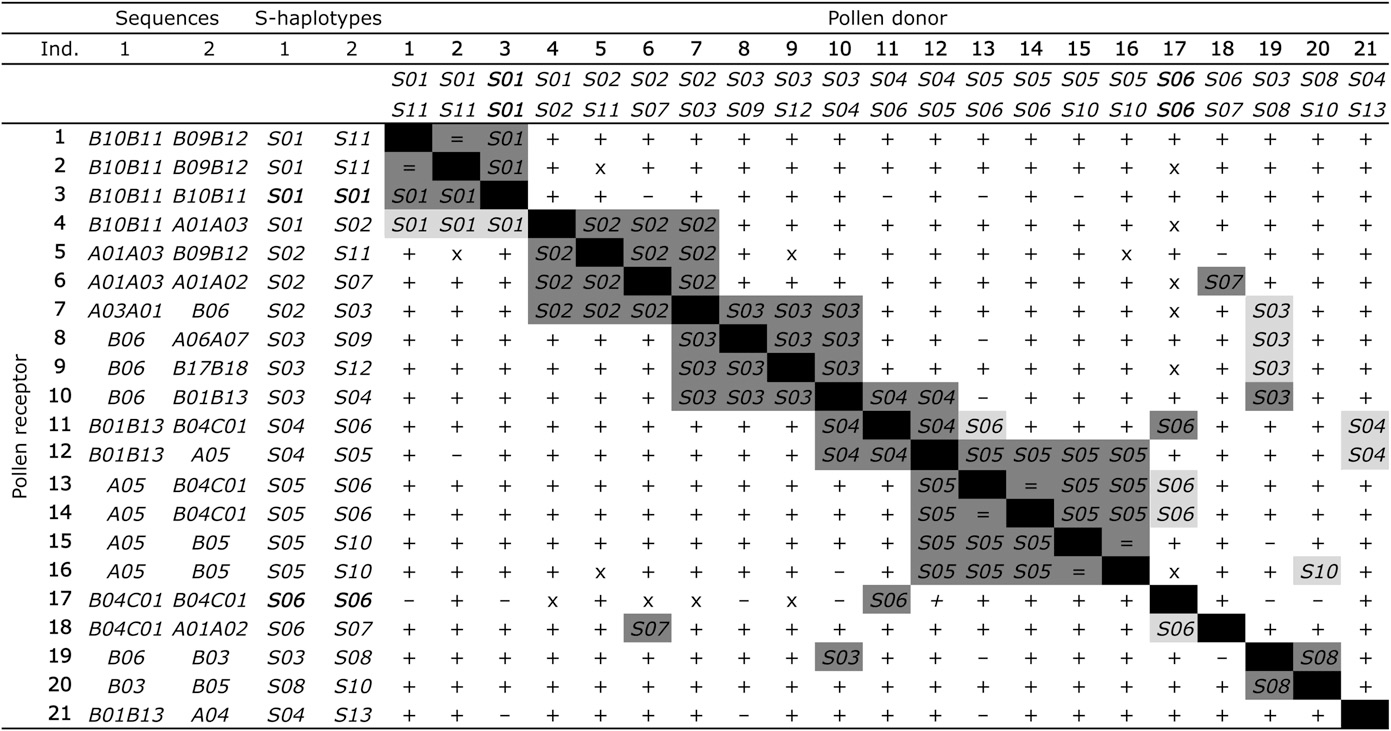
Results from controlled crosses realized among 21 individuals of collection F0 (Leducq *et al.* 2010) related with *S*-genotypes determined in this study. Linked groups of *SRK*-like sequences found in each individual are indicated in second and third columns; corresponding *S*-haplotypes are indicated in fourth and fifth columns. Black boxes represent unsuccessful self-pollinations; gray boxes represent reciprocal unsuccessful cross-pollinations, filled with the expressed *S*-haplotype when shared between crossed individuals (sign equal when to crossed individuals have identical genotypes). Light gray box represent nonreciprocal unsuccessful cross-pollinations. Successful cross-pollinations are represented by plus signs (+). Unsuccessful cross pollinations realized between individuals sharing no *S*-haplotypes are indicated by minus signs (-). Unrealized pollinations are represented by crosses (x).

**Figure 4:**
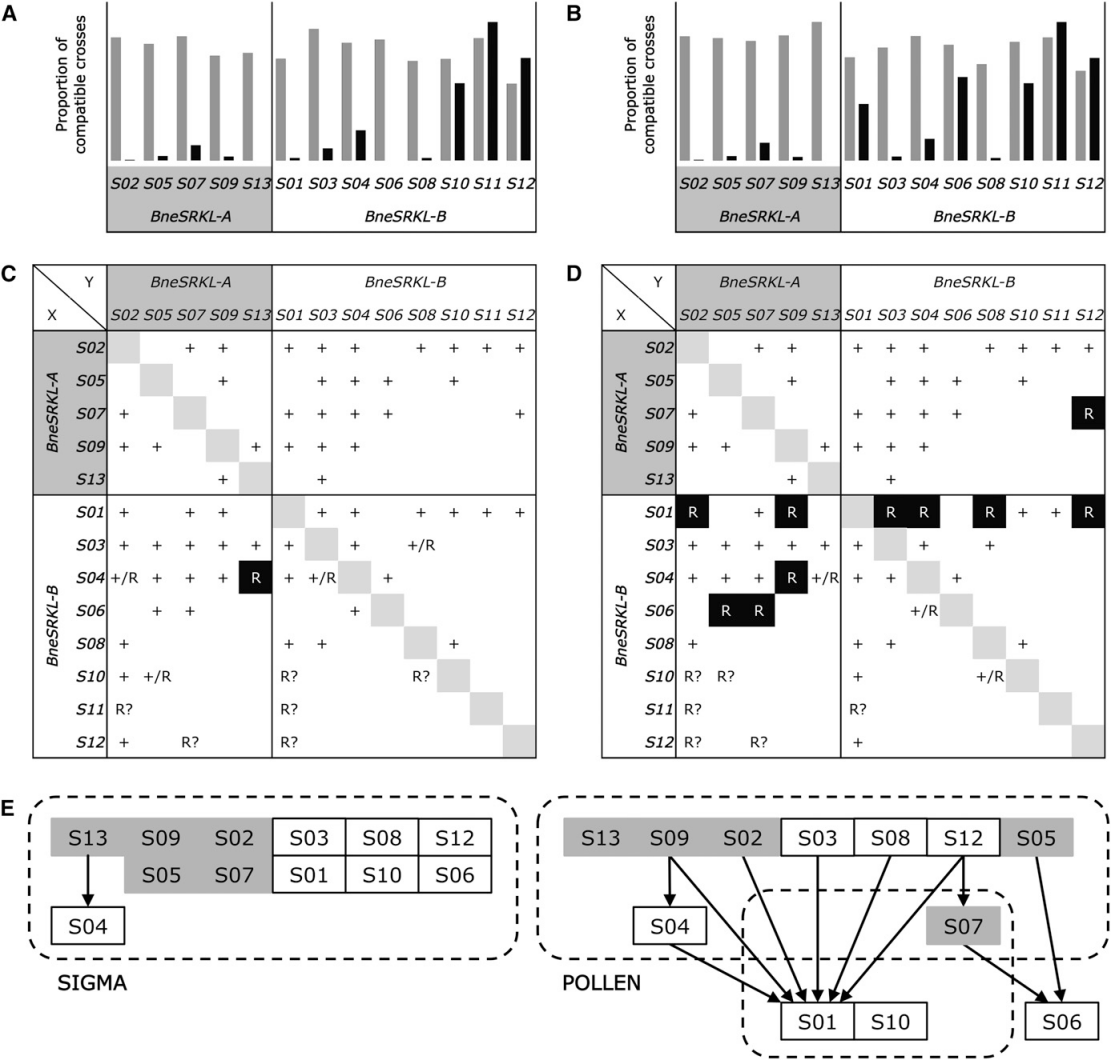
Dominance relationships determined by controlled pollinations between 13 S-haplotypes in *B. neustriaca*. Proportion of successful pollinations calculated for all crosses involving stigma (A) or pollen (B) of an individual with a given *S*-haplotype (x-axis). Crosses between individuals sharing or not sharing the haplotype are filled in black and gray, respectively. Pattern of SI expression for *S*-haplotypes involved in 35 possible genotypes in stigma (C) and pollen (D). Results are indicated for the haplotype X (in rows) as compared to haplotype Y (in columns) and report whether X was expressed (+) or not (R). Uncertain cases resulted from either the lack of evidence for X function (R?) or from inconsistencies between different individuals with the same genotype (+/R). (E) Dominance relationships among 12 S-haplotypes (S11 removed) in stigma (left) and pollen (right). Only nonambiguous cases were reported here. Dotted frames regroup haplotypes that are mostly codominant. Arrows indicate dominance relationships. Haplotypes from classes A and B are filed in gray and white respectively. See Figure 3, Figure S1, Figure S2, Figure S3, Figure S4, Figure S5, Figure S6, Figure S7, Figure S8, Figure S9, Figure S10, Figure S11, Figure S12, Figure S13, and Figure S14 for detailed crosses.

To confirm the link between these haplotypes and the SI phenotypes in the offspring and to validate it for other putative *S*-haplotypes, we performed 1472 additional controlled pollinations between individuals of collection F1 (Figure S1, Figure S2, Figure S3, Figure S4, Figure S5, Figure S6, Figure S7, Figure S8, Figure S9, Figure S10, Figure S11, Figure S12, Figure S13, and Figure S14). Figure 4 summarizes our results from all crosses between individuals of collections F0 and F1. A total of 71% of positive controls (243 crosses between individuals sharing no putative *S*-haplotype) yielded fruits *vs.* 9% of crosses between individuals sharing one putative *S*-haplotype (1229 crosses; Figure 4, A−D). These results confirmed the functional incompatibility types of the *S*-haplotypes previously identified as *S01−S08* (Figure S1, Figure S2, Figure S3, Figure S4, Figure S5, Figure S6, Figure S7, and Figure S8) and validate the function of four other putative *S*-haplotypes *S09* (*A06*/*A07*; Figure S9), *S10* (*B05*; Figure S10), *S12* (*B17*/*B18*; Figure S12) and *S13* (*A04*; Figure S13), but not *S11* (*B09*/*B12*; Figure S11). Successful positive controls between individuals sharing no common sequence attested that these aborted pollinations most probably resulted from incompatibility reactions (Figure 4, A and B). Compatibility results were almost the same for different plants with identical genotypes (but see Figure S10 for an exception). Most crosses between individuals carrying *S07* (*A01*/*A02*) and *S02* (*A01*/*A03*) were fully compatible, indicating that, despite sharing the *A01* sequence, these two haplotypes confer distinct incompatibility types (Figure S14). The low sequence diversity we found within *A01* sequences (one nonsynonymous nucleotide substitution separating sequences *A01a* and *A01b*) was not associated with whether the other sequence in the haplotype was *A02* or *A03* (Table 1 and Table S4).

### Patterns of dominance among *S*-haplotypes

In controlled pollinations among F0 individuals, some pairs sharing one *S*-haplotype were fully compatible, indicating the occurrence of dominance relationships between *S*-haplotypes, as typical for sporophytic SI (Figure 3). Dominance relationships were not identical between pollen and stigmas. For instance, pollen from individual 4, carrying alleles *S01* and *S02*, was compatible with individual 3, carrying two copies of allele *S01* (whose expression in stigmas was validated by unsuccessful pollination by other individuals having *S01*), but these individuals were incompatible when individual 3 was the pollen donor (Figure 3 and Figure 4, A−D). Thus, allele *S01* is expressed in stigma of individual 4 (and is thus either dominant or codominant with allele *S02*) but recessive to *S02* in pollen. The varied compatibility in pollen and stigmas among the *S*-haplotypes in crosses between individuals sharing one *S*-haplotype (combining F0 and F1 crosses) is shown in Figure 4, A−B. The average proportion of compatible crosses was lower for *S*-haplotypes of class *BneSRK-A* (3%) than for *BneSRK-B* (23%), and the difference was less pronounced in stigmas (3 and 18% for A and B classes, respectively; Figure 4A) than in pollen (3 and 28% for A and B classes, respectively; Figure 4B). In stigmas, *S*-haplotypes of class A were expressed in all genotypes, whereas there was at least one case of recessivity for a class B haplotype (Figure 4C). We observed many more cases of recessivity in pollen, at least 10 cases, mostly involving two class B *S*-haplotypes (*S01* and *S06*), which were not expressed in most genotypes tested (Figure 4D). Overall, codominance was more frequent in stigmas than in pollen and recessive haplotypes generally belonged to class B (Figure 4E).

### Analysis of polymorphism among *SRKL* sequences and test of diversifying selection

To test for positive selection resulting from negative frequency-dependent selection, we analyzed polymorphisms in the *SRKL* sequences. Diversity was found to be high among sequences of *BneSRKL* classes A and B at both synonymous (*π_S_* = 9.9–17.0%) and non-synonymous sites (*π_N_* = 7.8–9.8%; 528−465 nucleotide sites; Table 4, Figure 2); class C has a single sequence. PAML analysis yielded evidence for a class of sites evolving under diversifying selection for both classes A and B (Table 4). Using a cut-off at *P* = 0.95 in the Bayes empirical Bayes analysis, we detected 2 codons evolving under positive selection for class A and 3 codons for class B (Table 4 and Figure 2). All three positively selected codons of class B were within regions corresponding to the hypervariable regions of Brassica sequences (Nishio and Kusaba 2000), and the same was true for one of the two class A selected codons (Figure 2). Three of these sites were also previously detected as positively selected in other Brassicaceae (Table 4). The posterior mean *dN*/*dS* for positively selected sites in classes A and B were 4.110 and 2.915, respectively (Table 4). Only one sequence, *BneSRKL_B12*, had a premature stop codon; this sequence has a 1bp deletion that resulted in a reading frame shift.

**Table 4:** Analyses of nucleotide polymorphism and detection of positive selection in sequences within phylogenetic groups *BneSRKL_A* and *BneSRKL_B*

						Log Likelihood				Positively Selected Sites	
	n_seq_	n_sites_	π_S_	π_N_	π_N_/π_S_	M7	M8	-2ΔLn	*p*-Value	Sites*^a^*	Average dN/dS
*BneSRKL_A*	7	528	0.0993	0.0777	0.78	−1432	−1429	6.37	0.041	262N 295S*^b^*	4.110
*BneSRKL_B*	10	465	0.1705	0.0984	0.58	−1714	−1708	11.71	0.003	274D*^b^*^,^*^c^*	2.915
										276*^b^*^,^*^d^*	
										303V*^b^*^,^*^c^*	

Abbreviations: n_seq_, number of sequences analyzed; nsites, number of nucleotide sites; π_S_ and π_S_, nucleotide diversity at synonymous and non-synonymomus sites, respectively.

a *BoSRK60* used as reference.

b Sites in hypervariable regions.

c Sites also detected in Arabidopsis and Brassica.

d Site also detected in Arabidopsis.

## Discussion

### Molecular identification of *SRK*-like sequences linked to the SI phenotype in the tribe Biscutelleae

Here, we identified *SRK*-like sequences in *B. neustriaca* and demonstrated that these sequences segregate at a single locus and are tightly linked with the SI phenotype. Our results suggest that these *SRK*-like sequences are functional *SRK* alleles similar to those in the Brassicaceae SSI system previously described in Brassica (Kusaba *et al.* 1997; Sato *et al.* 2002), Capsella (Guo *et al.* 2009; Paetsch *et al.* 2006), Arabidopsis (Castric and Vekemans 2007; Kusaba *et al.* 2001; Schierup *et al.* 2001), and Arabis (Tedder *et al.* 2011) and not those of Leavenworthia, where the gene involved in stigma SI is not homologous to the *SRK* of Arabidopsis and Brassica (Chantha *et al.* 2013).

### *SRK* sequences in the Biscutella and Brassica lineages have several features in common

The two large monophyletic groups, A and B, of *B. neustriaca SRKL* sequences resemble the situation observed in Brassica outlined in the Introduction, where the two sequence groups represent dominant and recessive alleles. A demographic event such as a strong bottleneck may have caused the low *SRK* diversity in Brassica (Castric and Vekemans 2007; Edh *et al.* 2009). The *B. neustriaca* clusters, including the single sequence of group C, do not cluster with those of Brassica. Moreover, the reduction in the number of lineages in *B. neustriaca* appears to have been followed by allelic diversification of two of the three surviving *SRK* lineages, with evidence of strong positive selection (*dN*/*dS* in the range 2.92−4.11) of the same order of magnitude as inferred in Brassica (2.98; Castric and Vekemans 2007) and in Leavenworthia (3.49; Herman *et al.* 2012) and contrasting with lower estimates for *A. lyrata* and *A. halleri* (1.49 and 1.44, respectively) whose *S*-locus diversification seems to be much more ancient.

The duplicated *SRKL* sequences within *B. neustriaca S*-haplotypes also resemble the situation in Brassica, where *SRK* and *SLG* co-segregate at the *S*-locus (Nishio and Kusaba 2000; Takuno *et al.* 2008). Cosegregation of SI-related sequences also was recently observed in some genera of Papaveraceae, which have a gametophytic system (Paape *et al.* 2011), and in Solaneaceae a set of genetically linked F-box genes collectively determines the pollen phenotype (Kubo *et al.* 2010). We cannot currently determine whether the two *B. neustriaca SRKL* sequences together determine the pistil phenotype, or, a single *SRK* sequence, as in Brassica. In one case, however, we have evidence that a sequence is probably not involved in pistil recognition, since *BneSRKL-A01* is shared by two functionally distinct *S*-haplotypes, *S02* and *S07*. Hence, further functional investigations should focus on the other *BneSRKL* sequences, *A02* and *A03*, also carried by these haplotypes. Genomic studies are now needed to determine the structure and genomic localization of the *S*-locus, and to obtain the full coding sequences of both *SRK*-like copies within *S*-haplotypes, to determine whether both sequences have a functional kinase domain, or whether one of them consistently lacks this like Brassica *SLG*.

### Number of *S*-alleles and patterns of dominance

*B. neustriaca* is a narrow endemic species whose populations are highly disconnected, with restricted gene flow (Leducq *et al.* 2013). Within natural populations, individuals typically aggregate in high-density patches (J.-B. Leducq, personal observations). In our previous study, we showed that highly reduced diversity at the *S*-locus and low local density could greatly reduce maternal reproductive success (Leducq *et al.* 2010), *i.e.*, there is a *S*-Allee effect (Wagenius *et al.* 2007). Given that *B. neustriaca* has at least 13 functionally distinct *S*-alleles in the 21 individuals sampled in collection F0, it might appear unlikely that ecological events could reduce allelic diversity enough to cause an overall reduced reproductive success in natural populations.

Recessivity of the SI phenotype in *B. neustriaca* is commoner in pollen than in stigmas, where codominance is most frequent, as was also found in Brassica and Arabidopsis. The difference between dominance in pollen and stigmas is often interpreted as a consequence of the more intense sexual selection on the male than on the female function, because dominance in pollen allows a plant to minimize the chances of its pollen being rejected by stigmas from other plants (Schoen and Busch 2009). Interestingly, all recessive *S*-haplotypes except for one appear to belong to class *B*, suggesting that different dominance is associated with different *S*-haplotype lineages, as observed in Brassica (Nasrallah *et al.* 1991) and Arabidopsis (Llaurens *et al.* 2008; Prigoda *et al.* 2005).

Because negative frequency-dependent selection acting on *S*-allele diversity is expected to be partially relaxed for recessive *S*-haplotypes, they are expected to reach greater frequencies in populations than dominant ones (Billiard *et al.* 2007; Llaurens *et al.* 2008; Schierup *et al.* 1997). Consistent with this prediction, the two most recessive *S*-haplotypes found in our sample of 21 individuals (*S01* and *S06*) were the most frequent. It should now be tested whether this is also true in natural populations of *B. neustriaca*.

## Supplementary Material

Supporting Information
