# Supplementary material for: Self-Incompatibility in Brassicaceae: Identification and Characterization of SRK-Like Sequences Linked to the S-Locus in the Tribe Biscutelleae
Source: G3 (Bethesda). 2013 Dec 23;4(6):983–92. doi: 10.1534/g3.114.010843 (PMC4065267; doi:10.1534/g3.114.010843)
Supplement: Supporting Information [file supp_4_6_983__index.html]

Supporting Information 

# Self-Incompatibility in Brassicaceae: Identification and Characterization of *SRK*-Like Sequences Linked to the *S*-Locus in the Tribe Biscutelleae

## Supporting Information for Leducq *et al.*, 2014

**Files in this Data Supplement:**

- Supporting Information - Tables S1-S4 and Figures S1-S14 (PDF, 771 KB)
- Table S1 - Accession numbers of *SRK*-related sequences for Figure 1, from *Arabidopsis halleri* (*Aha*), *A. lyrata* (*Aly*), *A. thaliana* (*Ath*), *Brassica oleraceae* (*Bol*), *B. rapa* (*Bra*) and *Capsella grandiflora* (*Cgr*). (PDF, 140 KB)
- Table S2 - List and sequences of primers targeting candidate sequences of the pollen-recognition extracellular domain of the *SRK* gene in *Biscutella neustriaca*. (PDF, 156 KB)
- Table S3 - Result of test of deviation from Mendelian expectations in collection F1 (See Table 3 for genotypes). (PDF, 126 KB)
- Table S4 - Accession numbers of *SRK*-L sequences found in *Biscutella neustriaca*. (PDF, 152 KB)
- Figure S1 - Summary of cross-pollinations realized for individuals from collection F0 and F1 having *S*-haplotype *S01* (*B10-B11*). (PDF, 213 KB)
- Figure S2 - Summary of cross-pollinations realized for individuals from collection F0 and F1 having *S*-haplotype *S02* (*A01-A03*). (PDF, 185 KB)
- Figure S3 - Summary of cross-pollinations realized for individuals from collection F0 and F1 having *S*-haplotype *S03* (*B06*). (PDF, 186 KB)
- Figure S4 - Summary of cross-pollinations realized for individuals from collection F0 and F1 having *S*-haplotype *S04* (*B01-B13*). (PDF, 177 KB)
- Figure S5 - Summary of cross-pollinations realized for individuals from collection F0 and F1 having *S*-haplotype *S05* (*A05*). (PDF, 160 KB)
- Figure S6 - Summary of cross-pollinations realized for individuals from collection F0 and F1 having *S*-haplotype *S06* (*B04-C01*). (PDF, 155 KB)
- Figure S7 - Summary of cross-pollinations realized for individuals from collection F0 and F1 having *S*-haplotype *S07* (*A01-A02*). (PDF, 181 KB)
- Figure S8 - Summary of cross-pollinations realized for individuals from collection F0 and F1 having *S*-haplotype *S08* (*B03*). (PDF, 150 KB)
- Figure S9 - Summary of cross-pollinations realized for individuals from collection F0 and F1 having *S*-haplotype *S09* (*A06-A07*). (PDF, 161 KB)
- Figure S10 - Summary of cross-pollinations realized for individuals from collection F0 and F1 having *S*-haplotype *S10* (*B05*). (PDF, 153 KB)
- Figure S11 - Summary of cross-pollinations realized for individuals from collection F0 and F1 having *S*-haplotype *S11* (*B09-B12*). (PDF, 143 KB)
- Figure S12 - Summary of cross-pollinations realized for individuals from collection F0 and F1 having *S*-haplotype *S12* (*B17-B18*). (PDF, 146 KB)
- Figure S13 - Summary of cross-pollinations realized for individuals from collection F0 and F1 having *S*-haplotype *S13* (*A04*). (PDF, 143 KB)
- Figure S14 - Cross-pollinations between plants having haplotype *S02* and plants having haplotype *S07* are mostly successful, indicating that *S02* and *S07* are distinct functional *S*-haplotypes, while sharing sequence *A01* in common. (PDF, 169 KB)
