# Supplementary material for: Self-Incompatibility in Brassicaceae: Identification and Characterization of SRK-Like Sequences Linked to the S-Locus in the Tribe Biscutelleae
Source: G3 (Bethesda). 2013 Dec 23;4(6):983–92. doi: 10.1534/g3.114.010843 (PMC4065267; doi:10.1534/g3.114.010843)
Supplement: Supporting Information [file supp_4.6.983_010843SI.pdf]

G3· Genes | Genomes | Genetics

SELF-INCOMPATIBILITY IN BRASSICACEAE: IDENTIFICATION AND CHARACTERIZATION OF *SRK*-LIKE SEQUENCES LINKED TO THE *S*-LOCUS IN THE TRIBE BISCUTELLEAE

Jean-Baptiste Leducq*†, Célia C Gosset§†, Rita Gries†, Kevin Calin†, Éric Schmitt†, Vincent Castric† & Xavier Vekemans†.

*- *Institut de Biologie Intégrative et des Systèmes, Département de Biologie, PROTEO, Pavillon Charles-Eugène-Marchand, 1030 avenue de la Médecine - Université Laval - Québec (QC) G1V 0A6, Canada*
§- *Institut des Sciences de l'Évolution UMR 5554, CNRS, Université Montpellier 2, Place Eugène Bataillon, C.C. 065 - 34095 Montpellier cedex 05, France*
†- *Laboratoire Génétique et Evolution des Populations Végétales, CNRS UMR 8198, Université Lille1, F-59655 Villeneuve d'Ascq cedex, France*

Corresponding author: Jean-Baptiste Leducq (jean-baptiste.leducq.1@ulaval.ca)

DOI: 10.1534/g3.114.010843

Table S1 Accession numbers of *SRK*-related sequences for Figure 1, from *Arabidopsis halleri* (*Aha*), *A. lyrata* (*Aly*), *A. thaliana* (*Ath*), *Brassica oleraceae* (*Bol*), *B. rapa* (*Bra*) and *Capsella grandiflora* (*Cgr*).

Sequence	Accession number	*Sequence*	Accession number
AhaSRK03	EU075126	*BolSRK06*	M76647.1
AhaSRK04	DQ520278	*BolSRK07*	AB070624.1
AhaSRK11	EU075132	*BolSRK08*	AB054708.1
AhaSRK12	EU075133	*BolSRK11*	AB054709.1
AhaSRK13	EU075134	*BolSRK12*	AB180901.1
AhaSRK15	EU075136	*BolSRK13*	AB024420.2
AhaSRK20	EU075141	*BolSRK15*	AB180903.1
AhaSRK22	EU075143	*BolSRK16*	AB054710.1
Aly327420	XM_002872670.1	*BolSRK18*	AB032473.1
AlyARK3_946052	XM_002867806.1	*BolSRK23*	AB013720.1
AlySRK03	AF328992	*BolSRK24*	AB054712.1
AlySRK04	AF328994	*BolSRK25*	AB054713.1
AlySRK05	AF328990	*BolSRK29*	Z30211.1
AlySRK06	DQ520281	*BolSRK33*	AB054714.1
AlySRK08	AY186766	*BolSRK35*	AB054715.1
AlySRK09	DQ520282	*BolSRK36*	AB054716.1
AlySRK10	AY186767	*BolSRK38*	AB054717.1
AlySRK12	AY186769	*BolSRK39*	AB054718.1
AlySRK13	AF328993	*BolSRK45*	AB054719.1
AlySRK14	AY186770	*BolSRK50*	AB054720.1
AlySRK15	AY186771	*BolSRK51*	AB054721.1
AlySRK16	DQ520283	*BolSRK57*	AB054722.1
AlySRK18	DQ520284	*BolSRK58*	AB054723.1
AlySRK19	AF328998	*BolSRK60*	AB032474.1
AlySRK20	AF328995	*BolSRK62*	AB054724.1
AlySRK22	DQ520285	*BolSRK64*	AB054725.1
AlySRK23	AF328997	*BraARK03_013522*	AB041621.1
AlySRK25	DQ520286	*BraSRK46*	AB013718.2
AlySRK27	EU878017	*BraSRK54*	AB219162.1
AlySRK29	AY186776	*BraSRK60*	AB097116.1
AlySRK30	EU878018	*CgrSRK01*	DQ530637.1
AlySRK33	EU878019	*CgrSRK02*	DQ530638.1
AlySRK35	EU878021	*CgrSRK03*	DQ530639.1
AlySRK36	DQ520288	*CgrSRK04*	DQ530640.1
AlySRK37	DQ520289	*CgrSRK05*	DQ530641.1
AlySRK38	EU165341	*CgrSRK06*	DQ530642.1
AlySRK39	EU878022	*CgrSRK07*	EF530735.1
AlySRK43	EU878024	*CgrSRK09*	FJ649955.1
AlySRK44	EU878025	*CgrSRK11*	FJ649956.1
AthSRKC	KC207414.1	*CgrSRK14*	FJ649958.1
BolSRK01	AB054706.1	*CgrSRK18*	FJ649953.1
BolSRK02	AJ306588.1	*CgrSRK24*	FJ649962.1
BolSRK03	X79432.1	*CgrSRK37*	FJ649959.1
BolSRK05	Y18259.1	*CgrSRK38*	FJ649960.1

Table S2 List and sequences of primers targeting candidate sequences of the pollen-recognition extracellular domain of the *SRK* gene in *Biscutella neustriaca*. Tm indicates the optimized hybridization temperature during PCR reaction. When the number of PCR cycles used is different from 35 cycles, it is indicated after Tm in parenthesis. Generalist primers amplify several sequences belonging to the same sequences class.

Class	F-specific primer		R-specific primer		Tm (°C)	Sequence(s) amplified
Bne327420L	X-F001	GTACCGGAGATGCGGAAGT	X-R540	GAGCTCTCCGGACCAAATTA	60	1 and 2
BneSRKA	A01-F142	AGGTGGAATTCGACGTTAAA	A01-R558	GTAATTACTGATATCCAATA	52	1
	A02-F277	TTTACGAAGAGGAATCCGGT	SLGR		50	2
	A03-F074	TTAAACTCACCAGCCCAGAAT	A03-R421	TATCCACAGTTGCCGCCCTTG	62	3
	A04-F068	ACACTTTTCAACTCACCAAGCGA	SLGR		59	4 and 5
	A06-F238	GACATGGACACTTCACCGAT	A06-R514	CAGACCCATTATTTCGGACA	62	6
	A-F020	GGGAACACATGGTTTCCAAC	SLGR		50	Generalist *A*
	A-F300	GTGGGAAAGGACAAATGGAA	SLGR		60	
	A-F294	GSWTGAGTGGGAAAKGRCAA	A-R461	CYGCGACATYTTTCCTCACA	60	
	A-F330	TGTGAGGAAGACACGGTTGA	SLGR		50	Generalist *A* and *C01*
BneSRKB	B01-F079	ATGACCAACCAAACCATACAG	SLGR	ATCTGACATAAAGATCTTGACC	52	1
	B-F075	CTTCATGACCAACCAAAGCA	B-R495	CGCATTCGCAAATCCTCTAC	60	3
	B04-F159	ATATGGATGGAGACAGATGT	B04-R371	GTAAAGCCATCTCCCCTGTA	60	4
	B05-F112	GTGAGTTTTACGGGGTCTTTCG	B05-R405	TGTAGTATCCGGCAACTTAA	60	5
	B06-F276	GTTTGATCCTAGAAATCCACAT	B06-R463	CTTTAAGGCACCTCTTCTCA	60	6
	B09-F147	GATTCCGATATCAGATGACT	B-R564	WRCATAATTCCGAATATCAA	50	9
	B10-F238	GACATGAACACGTCACCGGT	B10-R452	TTCTTACATTCTTTCGTATCA	52	10
	B11-F153	GACATCATATGAATGGAGCCCG	B-R539	AKCAYTYCYKTCCAAATAAC	52	11
	B12-F110	CTGTGAGTTTTACGGGCTTT	B12-R408	TGTAGTGTCCGGCAGCTTTACC	63	12
	B17-F115	ACTTTCTGGGGGTATTTAAATA	B-R388	TTATCTTCCTCARCCGSGTA	58 (40c)	17
	B-F055	GATGARATCGCTTACACGTT	B-R564	WRCATAATTCCGAATATCAA	54	Generalist *B*
	B-F064	GCTTACACGTTCYTMATGACC	B-R388	TTATCTTCCTCARCCGSGTA	50	
			B-R539	AKCAYTYCYKTCCAAATAAC	60	
BneSRKC	C-F001	GCTGCAGAGGTGACAAGTTTT	C-R173	GGATGGGTTGTCTGATTTGG	56	1

Table S3 Result of test of deviation from Mendelian expectations in collection F1 (See Table 3 for genotypes). For each cohort size *n*, 100,000 cohorts were simulated according to two scenarios: (1) assuming that both parents were heterozygous at the *S*-locus and (2) assuming that one parent was homozygous and one parent heterozygous. For each cohort, the following information is shown: number of seedlings (cohort size), name of F0 parental individuals, absolute expected frequency of each genotype in the cohort and difference (deviation) between observed and expected frequencies under each scenario.

Seedling collection	n[a]	Parents[b]		Expected genotype frequency in F1 (SD)[c]		Deviation from expected genotype frequency (associated *p*-value)[d]					
						(1) He[e]				(2) Ho[f]	
		1	2	(1) He[e]	(2) Ho[f]	1	2	3	4	1	2
F1o	7	3	6	1.75 (1.15)	3.50 (1.32)	-2.25* (0.01)	-1.25 (0.07)	–	–	-0.5 (0.23)	0.5 (0.50)
	13	3	7	3.25 (1.56)	6.50 (1.8)	-3.75* (0.01)	-2.75* (0.03)	–	–	-0.5 (0.30)	0.5 (0.50)
	5	8	4	1.25 (0.97)	2.50 (1.12)	-0.75 (0.11)	0.25 (0.37)	-0.75 (0.10)	–	–	–
	7	8	6	1.75 (1.15)	3.50 (1.33)	0.75 (0.45)	-1.25 (0.07)	-1.25 (0.07)	–	–	–
	21	9	4	5.25 (1.98)	10.50 (2.29)	1.25 (0.37)	4.25* (0.02)	-4.75* (0.01)	-0.75 (0.26)	–	–
	7	9	6	1.75 (1.14)	3.50 (1.32)	-1.25 (0.07)	-0.25 (0.24)	-0.25 (0.24)	–	–	–
	6	10	4	1.50 (1.06)	3.00 (1.22)	0.5 (0.47)	-2.5* (0.01)	0.5 (0.47)	–	–	–
	16	10	6	4.00 (1.73)	8.00 (2.00)	2 (0.20)	-2 (0.08)	-2 (0.08)	2 (0.20)	–	–
F1c	14	4	20	3.50 (1.62)	7.00 (1.87)	0.5 (0.48)	1.5 (0.28)	-0.5 (0.26)	-1.5 (0.11)	–	–
	30	12	8	7.50 (2.37)	15.00 (2.74)	0.5 (0.49)	1.5 (0.35)	1.5 (0.35)	-3.5 (0.05)	–	–
	30	17	18	7.50 (2.37)	15.00 (2.73)	-5.5* (0.01)	-9.5*** (0.00)	–	–	2 (0.29)	-2 (0.18)
	50	18	1	12.50 (3.06)	25.00 (3.54)	1.5 (0.38)	-2.5 (0.16)	3.5 (0.16)	-2.5 (0.16)	–	–
	14	21	8	3.50 (1.62)	7 .00 (1.87)	-0.5 (0.26)	2.5 (0.10)	-1.5 (0.11)	-0.5 (0.26)	–	–

a cohort size
b F0 collection
c expected genotype frequency in F1 collection following symbols are associated with significance of *p*-values: "***": $p \leq 0.001$; "*"; $p \leq 0.05$.
d expected frequency : see table 3
e assuming that both parents are heterozygous at the *S*-locus
f assuming that parent 1 is homozygous and that parent 2 is heterozygous at the *S*-locus - Not applied for cohorts with more than two genotypes

Table S4 Accession numbers of *SRK*-L sequences found in *Biscutella neustriaca*.

Sequence	Accession number
BneSRKL_A01a	KF905296
BneSRKL_A01b	KF905297
BneSRKL_A02	KF905298
BneSRKL_A03	KF905299
BneSRKL_A04	KF905300
BneSRKL_A05	KF905301
BneSRKL_A06	KF905302
BneSRKL_A07	KF905303
BneSRKL_B01	KF905304
BneSRKL_B03	KF905305
BneSRKL_B04	KF905306
BneSRKL_B05	KF905307
BneSRKL_B06	KF905308
BneSRKL_B09	KF905309
BneSRKL_B10	KF905310
BneSRKL_B11	KF905311
BneSRKL_B12	KF905312
BneSRKL_B13	KF905313
BneSRKL_B17	KF905314
BneSRKL_B18	KF905315
BneSRKL_C01	KF905295
BneARK3L_1	KF905320
BneARK3L_2	KF905321
BneARK3L_3	KF905322
BneARK3L_4	KF905323
BneARK3L_5	KF905324
Bne327420L_1	KF905316
Bne327420L_2	KF905317
Bne327420L_3	KF905318
Bne327420L_4	KF905319

S01

				Pollen donors F0			Pollen donors F1									
	S-haplotypes 1			*S01*	*S01*	*S01*	*S01*	*S01*	*S01*	*S01*	*S01*	*S01*	*S01*	Controls	S-shared vs. Controls	Expressed in stigma ?
	S-haplotypes 2			*S02*	*S11*	*S01*	*S07*	*S09*	*S04*	*S08*	*S10*	*S03*	*S12*			
	1	2	Plants	1	2	1	2	1	2	2	3	4	1			
Pollen receptors (stigma) F0	*S01*	*S02*	1	0/5	1/10	0/5	/	/	/	/	/	/	/	59/65		yes
F0	*S01*	*S11*	2	9/10**	0/10	0/10*	/	/	/	/	/	/	/	153/185		yes*
F0	*S01*	*S01*	1	4/5**	0/10*	0/5	0/10*	3/4**	3/4**	9/10**	0/5*	15/23**	5/5**	55/90		yes*
F1	*S01*	*S07*	2	/	/	0/9	/	/	/	/	/	/	/	4/5		yes
F1	*S01*	*S09*	1	/	/	0/5	/	/	/	/	/	/	/	5/5		yes
F1	*S01*	*S04*	3	/	/	1/10*	/	/	3/10**	/	/	/	/	3/5		yes*
F1	*S01*	*S08*	2	/	/	0/9	/	/	/	/	/	/	/	NA	NA	yes
F1	*S01*	*S10*	3	/	/	0/5	/	/	/	/	/	/	/	1/5		yes
F1	*S01*	*S03*	4	/	/	0/20	/	/	/	/	/	/	/	13/15		yes
F1	*S01*	*S12*	1	/	/	0/5	/	/	/	/	/	/	/	4/5		yes
Controls				59/65	136/164	58/85	8/10	5/5	5/5	NA	3/5	2/14	3/4			
S-shared *vs.* Controls										NA						
Expressed in pollen ?				no**	yes	yes	yes	no**	no**	no**	yes	no**	no**			

Figure S1 Summary of cross-pollinations realized for individuals from collection F0 and F1 having *S*-haplotype *S01* (*B10-B11*). For each tested cross-pollination between individuals having at least *S01* in common (*S*-shared crosses), pollen donor is shown in line and pollen receptor (stigma) in column. The number of plants having the same genotype used for cross-pollinations is shown next to genotypes. Slash bars indicate that no cross-pollination was realized for the given combination. For realized cross-pollinations, the number of successful pollinations over the number of realized pollinations is indicated. Black cells indicate that most (>70%) of cross-pollinations were unsuccessful. Controls indicate cross-pollinations realized between the tested genotype and all other plants sharing no common *S*-haplotype with the tested genotype (in grey when the low proportion of successful pollination could results from low pollen efficiency/receptivity). Histograms indicate the proportion of successful cross-pollinations for *S*-shared crosses totalized by line or column (black; crosses between identical genotypes were not counted) and controls (grey). We considered that *S01* was expressed in either pollen (columns) or stigma (lines) when most of combinations gave more than 70% of unsuccessful pollinations. In complex cases, asterisks indicate what combinations of crosses were considered for each diagnostic of expression. Question marks (?) indicate that results were too ambiguous or insufficient to conclude (for instance in cases of recessive haplotypes for which no homozygous was available to test for haplotype expression). In some cases, different individuals with the same genotype gave different results and then the two possible conclusions were indicated. No positive control could be obtained for plants of genotype (*S01, S08*).

S02				Pollen donors													
				F0				F1									
	S-haplotypes		1	*S02*	*S02*	*S02*	*S02*	*S02*	*S02*	*S02*	*S02*	*S02*	*S02*	*S02*	Controls	S-shared vs. Controls	Expressed in stigma ?
			2	*S07*	*S03*	*S11*	*S01*	*S09*	*S04*	*S08*	*S10*	*S03*	*S01*	*S12*			
	1	2	Plants	1	1	1	1	3	3	2	2	5	4	4			
Pollen receptors (stigma) F0	*S02*	*S07*	1	0/5	0/5	0/5	0/5	0/5	0/5	/	/	/	/	/	72/75		yes
F0	*S02*	*S03*	1	0/5	0/5	0/5	0/5	/	/	/	/	/	/	/	51/60		yes
F0	*S02*	*S11*	1	0/5	0/5	0/5	0/5	/	/	/	/	/	/	/	55/65		yes
F0	*S02*	*S01*	1	0/5	0/5	0/5	0/5	/	/	/	/	/	/	/	59/65		yes
F1	*S02*	*S09*	3	0/5	/	/	/	/	/	/	/	0/60	/	/	7/14		yes
F1	*S02*	*S04*	3	0/5	/	/	/	/	/	/	/	0/20	1/44	/	5/5		yes
F1	*S02*	*S08*	2	/	/	/	/	/	/	/	0/20	/	/	/	5/5		yes
F1	*S02*	*S10*	2	/	/	/	/	/	/	0/20	/	/	/	/	23/30		yes
F1	*S02*	*S03*	5	/	/	/	/	0/48	0/19	/	/	/	/	1/55	11/15		yes
F1	*S02*	*S01*	4	/	/	/	/	/	1/39	/	/	/	/	/	8/15		yes
F1	*S02*	*S12*	4	/	/	/	/	/	/	/	/	0/78	/	/	7/10		yes
	Controls			54/73	57/60	56/70	59/65	11/15	5/5	5/5	15/18	5/10	8/19	10/10			
	S-shared *vs.* Controls																
	Expressed in pollen ?			yes	yes	yes	yes	yes	yes	yes	yes	yes	yes	yes			

Figure S2 Summary of cross-pollinations realized for individuals from collection F0 and F1 having *S*-haplotype *S02* (*A01-A03*). See Figure S1 for legend details.

S03				Pollen donors													
				F0					F1								
	S-haplotypes		1	*S03*	*S03*	*S03*	*S03*	*S03*	*S03*	*S03*	*S03*	*S03*	*S03*	*S03*	Controls	S-shared vs. Controls	Expressed in stigma ?
			2	*S02*	*S09*	*S04*	*S08*	*S12*	*S07*	*S02*	*S05*	*S04*	*S01*	*S13*			
	1	2	Plants	1	1	1	1	1	2	4	3	3	4	2			
Pollen receptors (stigma) F0	*S03*	*S02*	1	0/5	0/5*	0/5*	0/5*	0/5*	/	/	/	/	/	/	51/60		yes
F0	*S03*	*S09*	1	0/5*	0/5	0/5*	1/5*	0/5*	0/5	/	/	/	/	/	57/78		yes
F0	*S03*	*S04*	1	1/5**	0/5*	0/5	1/10*	0/5*	0/4	/	/	/	/	/	59/70		yes
F0	*S03*	*S08*	1	5/5**	2/5**	1/5*	0/5	4/5**	/	/	/	/	/	/	55/76		yes*/no**
F0	*S03*	*S12*	1	0/5*	0/5*	0/5*	0/5*	0/5	0/5	/	/	/	/	/	60/75		yes
F1	*S03*	*S07*	2	/	0/5	0/4	/	/	/	0/5	/	/	/	/	20/22		yes
F1	*S03*	*S02*	4	/	/	/	/	/	0/5	/	/	/	0/35	/	8/10		yes
F1	*S03*	*S05*	3	/	/	/	/	/	/	/	/	0/15	/	0/19	8/9		yes
F1	*S03*	*S04*	3	/	/	/	/	/	/	/	0/15	/	/	0/5	5/5		yes
F1	*S03*	*S01*	4	/	/	/	/	/	/	4/33	/	/	/	/	13/15		yes
F1	*S03*	*S13*	2	/	/	/	/	/	/	/	1/20	0/5	/	/	9/9		yes
	Controls			57/60	67/85	57/80	59/85	65/70	19/25	4/5	7/10	8/10	2/14	8/10			
	S-shared *vs.* Controls																
	Expressed in pollen ?			yes*	yes*	yes*	yes*	yes*	yes	yes	yes	yes	yes	yes			

Figure S3 Summary of cross-pollinations realized for individuals from collection F0 and F1 having *S*-haplotype *S03* (*B06*). See Figure S1 for legend details.

S04

	S-haplotypes 1	S-haplotypes 2	Plants	Pollen donors F0: *S04* *S05* (1)	F0: *S04* *S03* (1)	F0: *S04* *S06* (1)	F0: *S04* *S13* (1)	F1: *S04* *S07* (2)	F1: *S04* *S02* (3)	F1: *S04* *S09* (2)	F1: *S04* *S03* (3)	F1: *S04* *S01* (2)	Controls	S-shared vs. Controls	Expressed in stigma ?
Pollen receptors (stigma) F0	*S04*	*S05*	1	0/5	1/10*	1/7*	1/5*	/	/	/	/	/	47/65		yes*
F0	*S04*	*S03*	1	0/5*	0/5	0/5*	10/13**	/	/	/	/	/	59/70		yes*/no**
F0	*S04*	*S06*	1	0/5*	0/5*	0/5	0/5*	/	/	/	/	/	63/70		yes*
F0	*S04*	*S13*	1	3/5**	3/5**	3/5**	1/5	/	/	/	/	/	60/85		no**
F1	*S04*	*S07*	2	/	/	/	/	/	1/10	/	/	0/5*	9/29		yes
F1	*S04*	*S02*	3	/	/	/	/	0/10*	/	/	0/5*	3/10**	5/5		yes*/ no**
F1	*S04*	*S09*	2	/	/	/	/	/	/	/	4/20	/	8/10		yes
F1	*S04*	*S03*	3	/	/	/	/	/	0/5*	9/38**	/	/	5/5		yes*
F1	*S04*	*S01*	3	/	/	/	/	0/5	0/10	/	/	/	3/5		yes
Controls				59/65	57/80	56/69	82/90	16/25	5/5	5/10	8/10	5/5			
S-shared *vs.* Controls															
Expressed in pollen ?				yes*	yes*	yes*	yes*/ no**	yes	yes	no**	yes	yes*/ no**			

Figure S4 Summary of cross-pollinations realized for individuals from collection F0 and F1 having *S*-haplotype *S04* (*B01-B13*). See Figure S1 for legend details.

S05

		S-haplotypes			Pollen donors							
					F0			F1				
				1	*S05*	*S05*	*S05*	*S05*	*S05*	Controls	S-shared vs. Controls	Expressed in stigma ?
				2	*S04*	*S10*	*S06*	*S09*	*S03*			
		1	2	Plants	1	2	2	3	3			
Pollen receptors (stigma)	F0	*S05*	*S04*	1	0/5	0/10	1/15	/	/	47/65		yes
		S05	*S10*	2	0/10	0/20	1/25	/	/	113/140		yes
		S05	*S06*	2	1/15	0/20	1/25	/	/	124/140		yes
	F1	*S05*	*S09*	3	/	/	/	/	1/19	11/15		yes
		S05	*S03*	3	/	/	/	0/16	/	8/9		yes
		Controls			59/65	126/150	98/145	12/14	7/10			
		S-shared *vs.* Controls										
		Expressed in pollen ?			yes	yes	yes	yes	yes			

Figure S5 Summary of cross-pollinations realized for individuals from collection F0 and F1 having *S*-haplotype *S05* (*A05*). See Figure S1 for legend details.

S06				Pollen donors F0						
	S-haplotypes		1	*S06*	*S06*	*S06*	*S06*	Controls	S-shared vs. Controls	Expressed in stigma ?
			2	*S07*	*S05*	*S04*	*S06*			
	1	2	Plants	1	2	1	1			
Pollen receptors (stigma) F0	*S06*	*S07*	1	0/5	7/10**	5/5**	0/5*	65/75		yes*
	S06	*S05*	2	10/10**	1/25	9/10**	0/10*	124/140		yes*
	S06	*S04*	1	5/5**	8/15**	0/5	0/5*	63/70		yes*
	S06	*S06*	1	3/5**	5/10**	0/5*	0/5	37/75		?
	Controls			60/80	98/145	56/69	37/48			
	S-shared *vs.* Controls									
	Expressed in pollen ?			no**	no**	yes*/ no**	yes*			

Figure S6 Summary of cross-pollinations realized for individuals from collection F0 and F1 having *S*-haplotype *S06* (*B04-C01*). See Figure S1 for legend details.

S07

				Pollen donors F0		Pollen donors F1						
	S-haplotypes 1			*S07*	*S07*	*S07*	*S07*	*S07*	*S07*			
	S-haplotypes 2			*S02*	*S06*	*S04*	*S03*	*S01*	*S12*	Controls	S-shared vs. Controls	Expressed in stigma ?
	1	2	Plants	1	1	2	2	2	1			
Pollen receptors (stigma) F0	*S07*	*S02*	1	0/5	1/5	/	/	0/5	/	72/75		yes
Pollen receptors (stigma) F0	*S07*	*S06*	1	0/5	0/5	/	/	/	/	65/75		yes
Pollen receptors (stigma) F1	*S07*	*S04*	2	/	/	/	1/20	/	/	9/29		yes
Pollen receptors (stigma) F1	*S07*	*S03*	2	0/5	/	0/19	/	/	/	20/22		yes
Pollen receptors (stigma) F1	*S07*	*S01*	2	0/5*	/	/	/	/	8/18**	4/5		yes*
Pollen receptors (stigma) F1	*S07*	*S12*	1	/	/	/	/	0/9	/	5/5		yes
Controls				54/73	60/80	16/25	19/25	8/10	5/5			
S-shared *vs.* Controls												
Expressed in pollen ?				yes	yes	yes	yes	yes	no**			

Figure S7 Summary of cross-pollinations realized for individuals from collection F0 and F1 having *S*-haplotype *S07* (*A01-A02*). See Figure S1 for legend details.

S08

				Pollen donors F0		Pollen donors F1				
	S-haplotypes 1			*S08*	*S08*	*S08*	*S08*			
	S-haplotypes 2			*S10*	*S03*	*S02*	*S01*	Controls	S-shared vs. Controls	Expressed in stigma ?
	1	2	Plants	1	1	2	2			
Pollen receptors (stigma) F0	*S08*	*S10*	1	0/5	0/5	/	/	78/90		yes
Pollen receptors (stigma) F0	*S08*	*S03*	1	0/5	0/5	/	/	55/76		yes
Pollen receptors (stigma) F1	*S08*	*S02*	2	/	/	/	0/19	5/5		yes
Pollen receptors (stigma) F1	*S08*	*S01*	2	/	/	1/15	/	/	/	yes
	Controls			77/95	59/85	5/5	/			
	S-shared *vs.* Controls						/			
	Expressed in pollen ?			yes	yes	yes	yes			

Figure S8 Summary of cross-pollinations realized for individuals from collection F0 and F1 having *S*-haplotype *S08* (*B03*). See Figure S1 for legend details. No positive control could be obtained for plants of genotype (*S01,S08*).

S09

						Pollen donors							
						F1							
		S-haplotypes		1	*S09*	*S09*	*S09*	*S09*	*S09*	*S09*	Controls	S-shared vs. Controls	Expressed in stigma ?
				2	*S03*	*S02*	*S05*	*S04*	*S01*	*S13*			
		1	2	Plants	1	3	3	2	1	2			
Pollen receptors (stigma)	F0	*S09*	*S03*	1	/	0/5	/	/	/	/	51/78		yes
	F1	*S09*	*S02*	3	0/5	/	0/5	/	0/10	/	7/14		yes
		S09	*S05*	3	/	0/5	/	0/10	/	4/20	11/15		yes
		S09	*S04*	2	/	/	0/10	/	/	1/15	8/10		yes
		S09	*S01*	1	/	0/10	/	/	/	/	5/5		yes
		S09	*S13*	2	/	/	0/14	0/10	/	/	2/5		yes
		Controls			67/85	11/15	12/14	5/10	5/5	4/4			
		S-shared *vs.* Controls											
		Expressed in pollen ?			yes	yes	yes	yes	yes	yes			

Figure S9 Summary of cross-pollinations realized for individuals from collection F0 and F1 having *S*-haplotype *S09* (*A06-A07*). See Figure S1 for legend details.

S10				Pollen donors						
				F0		F1				
	S-haplotypes		1	*S10*	*S10*	*S10*	*S10*	Controls	S-shared vs. Controls	Expressed in stigma ?
			2	*S05*	*S08*	*S02*	*S01*			
	1	2	Plants	2	1	2	3			
Pollen receptors (stigma) F0	*S10*	*S05*	2	0/20	0/5*<br>5/5**	/	/	113/140		yes*/no**
Pollen receptors (stigma) F0	*S10*	*S08*	1	9/10**	0/5	/	/	78/90		?**
Pollen receptors (stigma) F1	*S10*	*S02*	2	/	/	/	1/20	23/30		yes
Pollen receptors (stigma) F1	*S10*	*S01*	3	/	/	14/25**	/	1/5		?**
	Controls			126/150	77/95	15/18	3/5			
	S-shared *vs.* Controls									
	Expressed in pollen ?			?**	yes*/<br>no**	?**	yes			

Figure S10 Summary of cross-pollinations realized for individuals from collection F0 and F1 having *S*-haplotype *S10* (*B05*). See Figure S1 for legend details.

S11					Pollen donors F0				
		S-haplotypes		1	*S11*	*S11*	Controls	S-shared vs. Controls	Expressed in stigma ?
				2	*S02*	*S01*			
		1	2	Plants	1	2			
Pollen receptors (stigma)	F0	*S11*	*S02*	1	0/5	4/5*	55/65		?
		S11	*S01*	2	5/5**	0/5	82/110		?
		Controls			56/70	68/85			
		S-shared *vs.* Controls							
		Expressed in pollen ?			?	?			

Figure S11 Summary of cross-pollinations realized for individuals from collection F0 and F1 having *S*-haplotype *S11* (*B09-B12*). See Figure S1 for legend details.

S12					Pollen donors F1					
		S-haplotypes		1	*S12*	*S12*	*S12*	Controls	S-shared vs. Controls	Expressed in stigma ?
				2	*S07*	*S02*	*S01*			
		1	2	Plants	1	4	1			
Pollen receptors (stigma)	F1	*S12*	*S07*	1		5/5		NA	NA	?
		S12	*S02*	4	5/5		0/5*	7/10		yes*
		S12	*S01*	1		5/5		4/5		?
		Controls			NA	10/10	3/4			
		S-shared *vs.* Controls			NA					
		Expressed in pollen ?			?	?	yes*			

Figure S12 Summary of cross-pollinations realized for individuals from collection F0 and F1 having *S*-haplotype *S12* (*B17*-*B18*). See Figure S1 for legend details.

Haplotype *S12* was not typed in collection F1 because of no available specific primers for *B17* or *B18*. The presence of *S12* in collection F1 was thus deduced from the segregation pattern in offspring of individual 9 of (genotype *S03*-*S12;* see Figure 3 and Table 3). In collection F1, we thus considered that *S12* was present when *S03* (*B06*) was absent.

S13

		S-haplotypes 1	S-haplotypes 2	Plants	Pollen donors F1: S13 / S09 (2 plants)	Pollen donors F1: S13 / S03 (2 plants)	Controls	S-shared vs. Controls	Expressed in stigma ?
Pollen receptors (stigma)	F1	*S13*	*S09*	2	/	0/20	2/5		yes
Pollen receptors (stigma)	F1	*S13*	*S03*	2	0/20	/	9/9		yes
Controls					4/4	8/10			
S-shared *vs.* Controls									
Expressed in pollen ?					yes	yes			

Figure S13 Summary of cross-pollinations realized for individuals from collection F0 and F1 having *S*-haplotype *S13* (*A04*).

S02 vs. S07					Pollen donnors											
					F0	F1										
		S-haplotypes		1	**S02**	**S07**	**S07**	**S07**	**S07**	**S02**	**S02**	**S02**	**S02**	**S02**	**S02**	**S02**
				2	**S07**	S04**	S03*	S01	S12***	S03*	S01	S09	S04**	S08	S10	S12
		1	2	Plants	1	2	2	2	1	5	4	3	3	2	2	4
Pollen receptors (stigma)	F0	**S02**	**S07**	1	/	/	/	0/5	/	/	/	0/5	0/5	/	/	/
	F1	**S07**	S04**	2	/	/	1/20	/	/	1/5	8/19	/	1/10**	/	/	/
		**S07**	S03*	2	0/5	0/19	/	/	/	0/5*	/	/	5/5	/	7/8	5/5
		**S07**	S01	2	0/5	/	/	/	8/18***	4/5	/	/	/	/	/	/
		**S07**	S12	1	/	/	/	0/9	/	/	/	/	/	/	/	5/5
		**S02**	S03*	5	/	3/5	0/5*	8/10	/	/	/	0/48	0/19	/	/	1/55
		**S02**	S01	4	/	8/15	/	/	/	/	/	/	1/39	/	/	/
		**S02**	S09	3	0/5	/	/	/	/	0/60	/	/	/	/	/	/
		**S02**	S04**	3	0/5	0/10**	5/5	/	/	0/20	1/44	/	/	/	/	/
		**S02**	S08	2	/	/	/	/	/	/	/	/	/	/	0/20	/
		**S02**	S10	2	/	/	8/10	/	/	/	/	/	/	0/20	/	/
		**S02**	S12	4	/	/	2/5	/	5/5	0/78	/	/	/	/	/	/

Figure S14 Cross-pollinations between plants having haplotype *S02* and plants having haplotype *S07* are mostly successful, indicating that *S02* and *S07* are distinct functional *S*-haplotypes, while sharing sequence *A01* in common. See Figure S1 for legend details.
