# Supplementary material for: Self-Incompatibility in Brassicaceae: Identification and Characterization of SRK-Like Sequences Linked to the S-Locus in the Tribe Biscutelleae
Source: G3 (Bethesda). 2013 Dec 23;4(6):983–92. doi: 10.1534/g3.114.010843 (PMC4065267; doi:10.1534/g3.114.010843)
Supplement: Supporting Information [file supp_4.6.983_TableS1.pdf]

Table S1 Accession numbers of *SRK*-related sequences for Figure 1, from *Arabidopsis halleri* (*Aha*), *A. lyrata* (*Aly*), *A. thaliana* (*Ath*), *Brassica oleraceae* (*Bol*), *B. rapa* (*Bra*) and *Capsella grandiflora* (*Cgr*).

Sequence	Accession number	*Sequence*	Accession number
AhaSRK03	EU075126	*BolSRK06*	M76647.1
AhaSRK04	DQ520278	*BolSRK07*	AB070624.1
AhaSRK11	EU075132	*BolSRK08*	AB054708.1
AhaSRK12	EU075133	*BolSRK11*	AB054709.1
AhaSRK13	EU075134	*BolSRK12*	AB180901.1
AhaSRK15	EU075136	*BolSRK13*	AB024420.2
AhaSRK20	EU075141	*BolSRK15*	AB180903.1
AhaSRK22	EU075143	*BolSRK16*	AB054710.1
Aly327420	XM_002872670.1	*BolSRK18*	AB032473.1
AlyARK3_946052	XM_002867806.1	*BolSRK23*	AB013720.1
AlySRK03	AF328992	*BolSRK24*	AB054712.1
AlySRK04	AF328994	*BolSRK25*	AB054713.1
AlySRK05	AF328990	*BolSRK29*	Z30211.1
AlySRK06	DQ520281	*BolSRK33*	AB054714.1
AlySRK08	AY186766	*BolSRK35*	AB054715.1
AlySRK09	DQ520282	*BolSRK36*	AB054716.1
AlySRK10	AY186767	*BolSRK38*	AB054717.1
AlySRK12	AY186769	*BolSRK39*	AB054718.1
AlySRK13	AF328993	*BolSRK45*	AB054719.1
AlySRK14	AY186770	*BolSRK50*	AB054720.1
AlySRK15	AY186771	*BolSRK51*	AB054721.1
AlySRK16	DQ520283	*BolSRK57*	AB054722.1
AlySRK18	DQ520284	*BolSRK58*	AB054723.1
AlySRK19	AF328998	*BolSRK60*	AB032474.1
AlySRK20	AF328995	*BolSRK62*	AB054724.1
AlySRK22	DQ520285	*BolSRK64*	AB054725.1
AlySRK23	AF328997	*BraARK03_013522*	AB041621.1
AlySRK25	DQ520286	*BraSRK46*	AB013718.2
AlySRK27	EU878017	*BraSRK54*	AB219162.1
AlySRK29	AY186776	*BraSRK60*	AB097116.1
AlySRK30	EU878018	*CgrSRK01*	DQ530637.1
AlySRK33	EU878019	*CgrSRK02*	DQ530638.1
AlySRK35	EU878021	*CgrSRK03*	DQ530639.1
AlySRK36	DQ520288	*CgrSRK04*	DQ530640.1
AlySRK37	DQ520289	*CgrSRK05*	DQ530641.1
AlySRK38	EU165341	*CgrSRK06*	DQ530642.1
AlySRK39	EU878022	*CgrSRK07*	EF530735.1
AlySRK43	EU878024	*CgrSRK09*	FJ649955.1
AlySRK44	EU878025	*CgrSRK11*	FJ649956.1
AthSRKC	KC207414.1	*CgrSRK14*	FJ649958.1
BolSRK01	AB054706.1	*CgrSRK18*	FJ649953.1
BolSRK02	AJ306588.1	*CgrSRK24*	FJ649962.1
BolSRK03	X79432.1	*CgrSRK37*	FJ649959.1
BolSRK05	Y18259.1	*CgrSRK38*	FJ649960.1
