# Supplementary material for: Self-Incompatibility in Brassicaceae: Identification and Characterization of SRK-Like Sequences Linked to the S-Locus in the Tribe Biscutelleae
Source: G3 (Bethesda). 2013 Dec 23;4(6):983–92. doi: 10.1534/g3.114.010843 (PMC4065267; doi:10.1534/g3.114.010843)
Supplement: Supporting Information [file supp_4.6.983_TableS2.pdf]

Table S2 List and sequences of primers targeting candidate sequences of the pollen-recognition extracellular domain of the *SRK* gene in *Biscutella neustriaca*. Tm indicates the optimized hybridization temperature during PCR reaction. When the number of PCR cycles used is different from 35 cycles, it is indicated after Tm in parenthesis. Generalist primers amplify several sequences belonging to the same sequences class.

Class		F-specific primer		R-specific primer	Tm (°C)	Sequence(s) amplified
Bne327420L	X-F001	GTACCGGAGATGCGGAAGT	X-R540	GAGCTCTCCGGACCAAATTA	60	1 and 2
BneSRKA	A01-F142	AGGTGGAATTCGACGTTAAA	A01-R558	GTAATTACTGATATCCAATA	52	1
	A02-F277	TTTACGAAGAGGAATCCGGT	SLGR		50	2
	A03-F074	TTAAACTCACCAGCCCAGAAT	A03-R421	TATCCACAGTTGCCGCCCTTG	62	3
	A04-F068	ACACTTTTCAACTCACCAAGCGA	SLGR		59	4 and 5
	A06-F238	GACATGGACACTTCACCGAT	A06-R514	CAGACCCATTATTTCGGACA	62	6
	A-F020	GGGAACACATGGTTTCCAAC	SLGR		50	Generalist *A*
	A-F300	GTGGGAAAGGACAAATGGAA	SLGR		60	
	A-F294	GSWTGAGTGGGAAAKGRCAA	A-R461	CYGCGACATYTTTCCTCACA	60	
	A-F330	TGTGAGGAAGACACGGTTGA	SLGR		50	Generalist *A* and *C01*
BneSRKB	B01-F079	ATGACCAACCAAACCATACAG	SLGR	ATCTGACATAAAGATCTTGACC	52	1
	B-F075	CTTCATGACCAACCAAAGCA	B-R495	CGCATTCGCAAATCCTCTAC	60	3
	B04-F159	ATATGGATGGAGACAGATGT	B04-R371	GTAAAGCCATCTCCCCTGTA	60	4
	B05-F112	GTGAGTTTTACGGGGTCTTTCG	B05-R405	TGTAGTATCCGGCAACTTAA	60	5
	B06-F276	GTTTGATCCTAGAAATCCACAT	B06-R463	CTTTAAGGCACCTCTTCTCA	60	6
	B09-F147	GATTCCGATATCAGATGACT	B-R564	WRCATAATTCCGAATATCAA	50	9
	B10-F238	GACATGAACACGTCACCGGT	B10-R452	TTCTTACATTCTTTCGTATCA	52	10
	B11-F153	GACATCATATGAATGGAGCCCG	B-R539	AKCAYTYCYKTCCAAATAAC	52	11
	B12-F110	CTGTGAGTTTTACGGGCTTT	B12-R408	TGTAGTGTCCGGCAGCTTTACC	63	12
	B17-F115	ACTTTCTGGGGGTATTTAAATA	B-R388	TTATCTTCCTCARCCGSGTA	58 (40c)	17
	B-F055	GATGARATCGCTTACACGTT	B-R564	WRCATAATTCCGAATATCAA	54	Generalist *B*
	B-F064	GCTTACACGTTCYTMATGACC	B-R388	TTATCTTCCTCARCCGSGTA	50	
			B-R539	AKCAYTYCYKTCCAAATAAC	60	
BneSRKC	C-F001	GCTGCAGAGGTGACAAGTTTT	C-R173	GGATGGGTTGTCTGATTTGG	56	1
