# Supplementary material for: Self-Incompatibility in Brassicaceae: Identification and Characterization of SRK-Like Sequences Linked to the S-Locus in the Tribe Biscutelleae
Source: G3 (Bethesda). 2013 Dec 23;4(6):983–92. doi: 10.1534/g3.114.010843 (PMC4065267; doi:10.1534/g3.114.010843)
Supplement: Supporting Information [file supp_4.6.983_TableS3.pdf]

Table S3 Result of test of deviation from Mendelian expectations in collection F1 (See Table 3 for genotypes). For each cohort size *n*, 100,000 cohorts were simulated according to two scenarios: (1) assuming that both parents were heterozygous at the *S*-locus and (2) assuming that one parent was homozygous and one parent heterozygous. For each cohort, the following information is shown: number of seedlings (cohort size), name of F0 parental individuals, absolute expected frequency of each genotype in the cohort and difference (deviation) between observed and expected frequencies under each scenario.

Seedling collection	n[a]	Parents[b]		Expected genotype frequency in F1 (SD)[c]		Deviation from expected genotype frequency (associated *p*-value)[d]					
						(1) He[e]				(2) Ho[f]	
		1	2	(1) He[e]	(2) Ho[f]	1	2	3	4	1	2
F1o	7	3	6	1.75 (1.15)	3.50 (1.32)	-2.25* (0.01)	-1.25 (0.07)	–	–	-0.5 (0.23)	0.5 (0.50)
	13	3	7	3.25 (1.56)	6.50 (1.8)	-3.75* (0.01)	-2.75* (0.03)	–	–	-0.5 (0.30)	0.5 (0.50)
	5	8	4	1.25 (0.97)	2.50 (1.12)	-0.75 (0.11)	0.25 (0.37)	-0.75 (0.10)	–	–	–
	7	8	6	1.75 (1.15)	3.50 (1.33)	0.75 (0.45)	-1.25 (0.07)	-1.25 (0.07)	–	–	–
	21	9	4	5.25 (1.98)	10.50 (2.29)	1.25 (0.37)	4.25* (0.02)	-4.75* (0.01)	-0.75 (0.26)	–	–
	7	9	6	1.75 (1.14)	3.50 (1.32)	-1.25 (0.07)	-0.25 (0.24)	-0.25 (0.24)	–	–	–
	6	10	4	1.50 (1.06)	3.00 (1.22)	0.5 (0.47)	-2.5* (0.01)	0.5 (0.47)	–	–	–
	16	10	6	4.00 (1.73)	8.00 (2.00)	2 (0.20)	-2 (0.08)	-2 (0.08)	2 (0.20)	–	–
F1c	14	4	20	3.50 (1.62)	7.00 (1.87)	0.5 (0.48)	1.5 (0.28)	-0.5 (0.26)	-1.5 (0.11)	–	–
	30	12	8	7.50 (2.37)	15.00 (2.74)	0.5 (0.49)	1.5 (0.35)	1.5 (0.35)	-3.5 (0.05)	–	–
	30	17	18	7.50 (2.37)	15.00 (2.73)	-5.5* (0.01)	-9.5*** (0.00)	–	–	2 (0.29)	-2 (0.18)
	50	18	1	12.50 (3.06)	25.00 (3.54)	1.5 (0.38)	-2.5 (0.16)	3.5 (0.16)	-2.5 (0.16)	–	–
	14	21	8	3.50 (1.62)	7 .00 (1.87)	-0.5 (0.26)	2.5 (0.10)	-1.5 (0.11)	-0.5 (0.26)	–	–

a cohort size

b F0 collection

c expected genotype frequency in F1 collection following symbols are associated with significance of *p*-values: "***": $p \leq 0.001$; "*"; $p \leq 0.05$.

d expected frequency : see table 3

e assuming that both parents are heterozygous at the *S*-locus

f assuming that parent 1 is homozygous and that parent 2 is heterozygous at the *S*-locus - Not applied for cohorts with more than two genotypes
