# Supplementary material for: Self-Incompatibility in Brassicaceae: Identification and Characterization of SRK-Like Sequences Linked to the S-Locus in the Tribe Biscutelleae
Source: G3 (Bethesda). 2013 Dec 23;4(6):983–92. doi: 10.1534/g3.114.010843 (PMC4065267; doi:10.1534/g3.114.010843)
Supplement: Supporting Information [file supp_4.6.983_TableS4.pdf]

Table S4 Accession numbers of *SRK*-L sequences found in *Biscutella neustriaca*.

Sequence	Accession number
BneSRKL_A01a	KF905296
BneSRKL_A01b	KF905297
BneSRKL_A02	KF905298
BneSRKL_A03	KF905299
BneSRKL_A04	KF905300
BneSRKL_A05	KF905301
BneSRKL_A06	KF905302
BneSRKL_A07	KF905303
BneSRKL_B01	KF905304
BneSRKL_B03	KF905305
BneSRKL_B04	KF905306
BneSRKL_B05	KF905307
BneSRKL_B06	KF905308
BneSRKL_B09	KF905309
BneSRKL_B10	KF905310
BneSRKL_B11	KF905311
BneSRKL_B12	KF905312
BneSRKL_B13	KF905313
BneSRKL_B17	KF905314
BneSRKL_B18	KF905315
BneSRKL_C01	KF905295
BneARK3L_1	KF905320
BneARK3L_2	KF905321
BneARK3L_3	KF905322
BneARK3L_4	KF905323
BneARK3L_5	KF905324
Bne327420L_1	KF905316
Bne327420L_2	KF905317
Bne327420L_3	KF905318
Bne327420L_4	KF905319
