# Supplementary material for: Self-Incompatibility in Brassicaceae: Identification and Characterization of SRK-Like Sequences Linked to the S-Locus in the Tribe Biscutelleae
Source: G3 (Bethesda). 2013 Dec 23;4(6):983–92. doi: 10.1534/g3.114.010843 (PMC4065267; doi:10.1534/g3.114.010843)
Supplement: Supporting Information [file supp_4.6.983_FigureS1.pdf]

S01

				Pollen donors F0			F1									
	S-haplotypes		1	*S01*	*S01*	*S01*	*S01*	*S01*	*S01*	*S01*	*S01*	*S01*	*S01*	Controls	S-shared vs. Controls	Expressed in stigma ?
			2	*S02*	*S11*	*S01*	*S07*	*S09*	*S04*	*S08*	*S10*	*S03*	*S12*			
	1	2	Plants	1	2	1	2	1	2	2	3	4	1			
Pollen receptors (stigma) F0	*S01*	*S02*	1	0/5	1/10	0/5	/	/	/	/	/	/	/	59/65		yes
F0	*S01*	*S11*	2	9/10**	0/10	0/10*	/	/	/	/	/	/	/	153/185		yes*
F0	*S01*	*S01*	1	4/5**	0/10*	0/5	0/10*	3/4**	3/4**	9/10**	0/5*	15/23**	5/5**	55/90		yes*
F1	*S01*	*S07*	2	/	/	0/9	/	/	/	/	/	/	/	4/5		yes
F1	*S01*	*S09*	1	/	/	0/5	/	/	/	/	/	/	/	5/5		yes
F1	*S01*	*S04*	3	/	/	1/10*	/	/	3/10**	/	/	/	/	3/5		yes*
F1	*S01*	*S08*	2	/	/	0/9	/	/	/	/	/	/	/	NA	NA	yes
F1	*S01*	*S10*	3	/	/	0/5	/	/	/	/	/	/	/	1/5		yes
F1	*S01*	*S03*	4	/	/	0/20	/	/	/	/	/	/	/	13/15		yes
F1	*S01*	*S12*	1	/	/	0/5	/	/	/	/	/	/	/	4/5		yes
	Controls			59/65	136/164	58/85	8/10	5/5	5/5	NA	3/5	2/14	3/4			
	S-shared *vs.* Controls									NA						
	Expressed in pollen ?			no**	yes	yes	yes	no**	no**	no**	yes	no**	no**			

Figure S1 Summary of cross-pollinations realized for individuals from collection F0 and F1 having *S*-haplotype *S01* (*B10-B11*). For each tested cross-pollination between individuals having at least *S01* in common (*S*-shared crosses), pollen donor is shown in line and pollen receptor (stigma) in column. The number of plants having the same genotype used for cross-pollinations is shown next to genotypes. Slash bars indicate that no cross-pollination was realized for the given combination. For realized cross-pollinations, the number of successful pollinations over the number of realized pollinations is indicated. Black cells indicate that most (>70%) of cross-pollinations were unsuccessful. Controls indicate cross-pollinations realized between the tested genotype and all other plants sharing no common *S*-haplotype with the tested genotype (in grey when the low proportion of successful pollination could results from low pollen efficiency/receptivity). Histograms indicate the proportion of successful cross-pollinations for *S*-shared crosses totalized by line or column (black; crosses between identical genotypes were not counted) and controls (grey). We considered that *S01* was expressed in either pollen (columns) or stigma (lines) when most of combinations gave more than 70% of unsuccessful pollinations. In complex cases, asterisks indicate what combinations of crosses were considered for each diagnostic of expression. Question marks (?) indicate that results were too ambiguous or insufficient to conclude (for instance in cases of recessive haplotypes for which no homozygous was available to test for haplotype expression). In some cases, different individuals with the same genotype gave different results and then the two possible conclusions were indicated. No positive control could be obtained for plants of genotype (*S01, S08*).
