# Supplementary material for: Self-Incompatibility in Brassicaceae: Identification and Characterization of SRK-Like Sequences Linked to the S-Locus in the Tribe Biscutelleae
Source: G3 (Bethesda). 2013 Dec 23;4(6):983–92. doi: 10.1534/g3.114.010843 (PMC4065267; doi:10.1534/g3.114.010843)
Supplement: Supporting Information [file supp_4.6.983_FigureS2.pdf]

S02

	S-haplotypes 1	S-haplotypes 2	Plants	Pollen donors F0 S02 S07 (1)	F0 S02 S03 (1)	F0 S02 S11 (1)	F0 S02 S01 (1)	F1 S02 S09 (3)	F1 S02 S04 (3)	F1 S02 S08 (2)	F1 S02 S10 (2)	F1 S02 S03 (5)	F1 S02 S01 (4)	F1 S02 S12 (4)	Controls	S-shared vs. Controls	Expressed in stigma ?
Pollen receptors (stigma) F0	*S02*	*S07*	1	0/5	0/5	0/5	0/5	0/5	0/5	/	/	/	/	/	72/75		yes
F0	*S02*	*S03*	1	0/5	0/5	0/5	0/5	/	/	/	/	/	/	/	51/60		yes
F0	*S02*	*S11*	1	0/5	0/5	0/5	0/5	/	/	/	/	/	/	/	55/65		yes
F0	*S02*	*S01*	1	0/5	0/5	0/5	0/5	/	/	/	/	/	/	/	59/65		yes
F1	*S02*	*S09*	3	0/5	/	/	/	/	/	/	/	0/60	/	/	7/14		yes
F1	*S02*	*S04*	3	0/5	/	/	/	/	/	/	/	0/20	1/44	/	5/5		yes
F1	*S02*	*S08*	2	/	/	/	/	/	/	/	0/20	/	/	/	5/5		yes
F1	*S02*	*S10*	2	/	/	/	/	/	/	0/20	/	/	/	/	23/30		yes
F1	*S02*	*S03*	5	/	/	/	/	0/48	0/19	/	/	/	/	1/55	11/15		yes
F1	*S02*	*S01*	4	/	/	/	/	/	1/39	/	/	/	/	/	8/15		yes
F1	*S02*	*S12*	4	/	/	/	/	/	/	/	/	0/78	/	/	7/10		yes
Controls				54/73	57/60	56/70	59/65	11/15	5/5	5/5	15/18	5/10	8/19	10/10			
S-shared *vs.* Controls																	
Expressed in pollen ?				yes	yes	yes	yes	yes	yes	yes	yes	yes	yes	yes			

Figure S2 Summary of cross-pollinations realized for individuals from collection F0 and F1 having *S*-haplotype *S02* (*A01-A03*). See Figure S1 for legend details.
