# Supplementary material for: Self-Incompatibility in Brassicaceae: Identification and Characterization of SRK-Like Sequences Linked to the S-Locus in the Tribe Biscutelleae
Source: G3 (Bethesda). 2013 Dec 23;4(6):983–92. doi: 10.1534/g3.114.010843 (PMC4065267; doi:10.1534/g3.114.010843)
Supplement: Supporting Information [file supp_4.6.983_FigureS3.pdf]

S03

				Pollen donors													
				F0					F1								
	S-haplotypes		1	*S03*	*S03*	*S03*	*S03*	*S03*	*S03*	*S03*	*S03*	*S03*	*S03*	*S03*	Controls	S-shared vs. Controls	Expressed in stigma ?
			2	*S02*	*S09*	*S04*	*S08*	*S12*	*S07*	*S02*	*S05*	*S04*	*S01*	*S13*			
	1	2	Plants	1	1	1	1	1	2	4	3	3	4	2			
Pollen receptors (stigma) F0	*S03*	*S02*	1	0/5	0/5*	0/5*	0/5*	0/5*	/	/	/	/	/	/	51/60		yes
	S03	*S09*	1	0/5*	0/5	0/5*	1/5*	0/5*	0/5	/	/	/	/	/	57/78		yes
	S03	*S04*	1	1/5**	0/5*	0/5	1/10*	0/5*	0/4	/	/	/	/	/	59/70		yes
	S03	*S08*	1	5/5**	2/5**	1/5*	0/5	4/5**	/	/	/	/	/	/	55/76		yes*/no**
	S03	*S12*	1	0/5*	0/5*	0/5*	0/5*	0/5	0/5	/	/	/	/	/	60/75		yes
F1	*S03*	*S07*	2	/	0/5	0/4	/	/	/	0/5	/	/	/	/	20/22		yes
	S03	*S02*	4	/	/	/	/	/	0/5	/	/	/	0/35	/	8/10		yes
	S03	*S05*	3	/	/	/	/	/	/	/	/	0/15	/	0/19	8/9		yes
	S03	*S04*	3	/	/	/	/	/	/	/	0/15	/	/	0/5	5/5		yes
	S03	*S01*	4	/	/	/	/	/	/	4/33	/	/	/	/	13/15		yes
	S03	*S13*	2	/	/	/	/	/	/	/	1/20	0/5	/	/	9/9		yes
	Controls			57/60	67/85	57/80	59/85	65/70	19/25	4/5	7/10	8/10	2/14	8/10			
	S-shared *vs.* Controls																
	Expressed in pollen ?			yes*	yes*	yes*	yes*	yes*	yes	yes	yes	yes	yes	yes			

Figure S3 Summary of cross-pollinations realized for individuals from collection F0 and F1 having *S*-haplotype *S03* (*B06*). See Figure S1 for legend details.
