# Supplementary material for: Self-Incompatibility in Brassicaceae: Identification and Characterization of SRK-Like Sequences Linked to the S-Locus in the Tribe Biscutelleae
Source: G3 (Bethesda). 2013 Dec 23;4(6):983–92. doi: 10.1534/g3.114.010843 (PMC4065267; doi:10.1534/g3.114.010843)
Supplement: Supporting Information [file supp_4.6.983_FigureS4.pdf]

S04

	S-haplotypes 1	S-haplotypes 2	Plants	Pollen donors F0 *S04* *S05* (1)	F0 *S04* *S03* (1)	F0 *S04* *S06* (1)	F0 *S04* *S13* (1)	F1 *S04* *S07* (2)	F1 *S04* *S02* (3)	F1 *S04* *S09* (2)	F1 *S04* *S03* (3)	F1 *S04* *S01* (2)	Controls	S-shared vs. Controls	Expressed in stigma ?
Pollen receptors (stigma) F0	*S04*	*S05*	1	0/5	1/10*	1/7*	1/5*	/	/	/	/	/	47/65		yes*
F0	*S04*	*S03*	1	0/5*	0/5	0/5*	10/13**	/	/	/	/	/	59/70		yes*/no**
F0	*S04*	*S06*	1	0/5*	0/5*	0/5	0/5*	/	/	/	/	/	63/70		yes*
F0	*S04*	*S13*	1	3/5**	3/5**	3/5**	1/5	/	/	/	/	/	60/85		no**
F1	*S04*	*S07*	2	/	/	/	/	/	1/10	/	/	0/5*	9/29		yes
F1	*S04*	*S02*	3	/	/	/	/	0/10*	/	/	0/5*	3/10**	5/5		yes*/ no**
F1	*S04*	*S09*	2	/	/	/	/	/	/	/	4/20	/	8/10		yes
F1	*S04*	*S03*	3	/	/	/	/	/	0/5*	9/38**	/	/	5/5		yes*
F1	*S04*	*S01*	3	/	/	/	/	0/5	0/10	/	/	/	3/5		yes
Controls				59/65	57/80	56/69	82/90	16/25	5/5	5/10	8/10	5/5			
S-shared *vs.* Controls															
Expressed in pollen ?				yes*	yes*	yes*	yes*/ no**	yes	yes	no**	yes	yes*/ no**			

Figure S4 Summary of cross-pollinations realized for individuals from collection F0 and F1 having *S*-haplotype *S04* (*B01-B13*). See Figure S1 for legend details.
