# Supplementary material for: Self-Incompatibility in Brassicaceae: Identification and Characterization of SRK-Like Sequences Linked to the S-Locus in the Tribe Biscutelleae
Source: G3 (Bethesda). 2013 Dec 23;4(6):983–92. doi: 10.1534/g3.114.010843 (PMC4065267; doi:10.1534/g3.114.010843)
Supplement: Supporting Information [file supp_4.6.983_FigureS5.pdf]

S05

				Pollen donors F0			Pollen donors F1				
	S-haplotypes 1			*S05*	*S05*	*S05*	*S05*	*S05*	Controls	S-shared vs. Controls	Expressed in stigma ?
	S-haplotypes 2			*S04*	*S10*	*S06*	*S09*	*S03*			
	1	2	Plants	1	2	2	3	3			
Pollen receptors (stigma) F0	*S05*	*S04*	1	0/5	0/10	1/15	/	/	47/65		yes
Pollen receptors (stigma) F0	*S05*	*S10*	2	0/10	0/20	1/25	/	/	113/140		yes
Pollen receptors (stigma) F0	*S05*	*S06*	2	1/15	0/20	1/25	/	/	124/140		yes
Pollen receptors (stigma) F1	*S05*	*S09*	3	/	/	/	/	1/19	11/15		yes
Pollen receptors (stigma) F1	*S05*	*S03*	3	/	/	/	0/16	/	8/9		yes
Controls				59/65	126/150	98/145	12/14	7/10			
S-shared *vs.* Controls											
Expressed in pollen ?				yes	yes	yes	yes	yes			

Figure S5 Summary of cross-pollinations realized for individuals from collection F0 and F1 having *S*-haplotype *S05* (*A05*). See Figure S1 for legend details.
