# Supplementary material for: Self-Incompatibility in Brassicaceae: Identification and Characterization of SRK-Like Sequences Linked to the S-Locus in the Tribe Biscutelleae
Source: G3 (Bethesda). 2013 Dec 23;4(6):983–92. doi: 10.1534/g3.114.010843 (PMC4065267; doi:10.1534/g3.114.010843)
Supplement: Supporting Information [file supp_4.6.983_FigureS6.pdf]

S06				Pollen donors F0						
	S-haplotypes		1	*S06*	*S06*	*S06*	*S06*	Controls	S-shared vs. Controls	Expressed in stigma ?
			2	*S07*	*S05*	*S04*	*S06*			
	1	2	Plants	1	2	1	1			
Pollen receptors (stigma) F0	*S06*	*S07*	1	0/5	7/10**	5/5**	0/5*	65/75		yes*
	S06	*S05*	2	10/10**	1/25	9/10**	0/10*	124/140		yes*
	S06	*S04*	1	5/5**	8/15**	0/5	0/5*	63/70		yes*
	S06	*S06*	1	3/5**	5/10**	0/5*	0/5	37/75		?
	Controls			60/80	98/145	56/69	37/48			
	S-shared *vs.* Controls									
	Expressed in pollen ?			no**	no**	yes*/ no**	yes*			

Figure S6 Summary of cross-pollinations realized for individuals from collection F0 and F1 having *S*-haplotype *S06* (*B04-C01*). See Figure S1 for legend details.
