# Supplementary material for: Self-Incompatibility in Brassicaceae: Identification and Characterization of SRK-Like Sequences Linked to the S-Locus in the Tribe Biscutelleae
Source: G3 (Bethesda). 2013 Dec 23;4(6):983–92. doi: 10.1534/g3.114.010843 (PMC4065267; doi:10.1534/g3.114.010843)
Supplement: Supporting Information [file supp_4.6.983_FigureS7.pdf]

S07

				Pollen donors								
				F0		F1						
	S-haplotypes		1	*S07*	*S07*	*S07*	*S07*	*S07*	*S07*	Controls	S-shared vs. Controls	Expressed in stigma ?
			2	*S02*	*S06*	*S04*	*S03*	*S01*	*S12*			
	1	2	Plants	1	1	2	2	2	1			
Pollen receptors (stigma) F0	*S07*	*S02*	1	0/5	1/5	/	/	0/5	/	72/75		yes
Pollen receptors (stigma) F0	*S07*	*S06*	1	0/5	0/5	/	/	/	/	65/75		yes
Pollen receptors (stigma) F1	*S07*	*S04*	2	/	/	/	1/20	/	/	9/29		yes
Pollen receptors (stigma) F1	*S07*	*S03*	2	0/5	/	0/19	/	/	/	20/22		yes
Pollen receptors (stigma) F1	*S07*	*S01*	2	0/5*	/	/	/	/	8/18**	4/5		yes*
Pollen receptors (stigma) F1	*S07*	*S12*	1	/	/	/	/	0/9	/	5/5		yes
	Controls			54/73	60/80	16/25	19/25	8/10	5/5			
	S-shared *vs.* Controls											
	Expressed in pollen ?			yes	yes	yes	yes	yes	no**			

Figure S7 Summary of cross-pollinations realized for individuals from collection F0 and F1 having *S*-haplotype *S07* (*A01-A02*). See Figure S1 for legend details.
