# Supplementary material for: Self-Incompatibility in Brassicaceae: Identification and Characterization of SRK-Like Sequences Linked to the S-Locus in the Tribe Biscutelleae
Source: G3 (Bethesda). 2013 Dec 23;4(6):983–92. doi: 10.1534/g3.114.010843 (PMC4065267; doi:10.1534/g3.114.010843)
Supplement: Supporting Information [file supp_4.6.983_FigureS8.pdf]

S08

Pollen receptors (stigma)				Pollen donors: F0		F1				
	S-haplotypes		1	*S08*	*S08*	*S08*	*S08*	Controls	S-shared vs. Controls	Expressed in stigma ?
			2	*S10*	*S03*	*S02*	*S01*			
	1	2	Plants	1	1	2	2			
F0	*S08*	*S10*	1	0/5	0/5	/	/	78/90		yes
F0	*S08*	*S03*	1	0/5	0/5	/	/	55/76		yes
F1	*S08*	*S02*	2	/	/	/	0/19	5/5		yes
F1	*S08*	*S01*	2	/	/	1/15	/	/	/	yes
	Controls			77/95	59/85	5/5	/			
	S-shared *vs.* Controls						/			
	Expressed in pollen ?			yes	yes	yes	yes			

Figure S8 Summary of cross-pollinations realized for individuals from collection F0 and F1 having *S*-haplotype *S08* (*B03*). See Figure S1 for legend details. No positive control could be obtained for plants of genotype (*S01,S08*).
