# Supplementary material for: Self-Incompatibility in Brassicaceae: Identification and Characterization of SRK-Like Sequences Linked to the S-Locus in the Tribe Biscutelleae
Source: G3 (Bethesda). 2013 Dec 23;4(6):983–92. doi: 10.1534/g3.114.010843 (PMC4065267; doi:10.1534/g3.114.010843)
Supplement: Supporting Information [file supp_4.6.983_FigureS9.pdf]

S09

				Pollen donors:	F0	F1							
				S-haplotypes 1	*S09*	*S09*	*S09*	*S09*	*S09*	*S09*			
				S-haplotypes 2	*S03*	*S02*	*S05*	*S04*	*S01*	*S13*	Controls	S-shared vs. Controls	Expressed in stigma ?
		1	2	Plants	1	3	3	2	1	2			
Pollen receptors (stigma)	F0	*S09*	*S03*	1	/	0/5	/	/	/	/	51/78		yes
	F1	*S09*	*S02*	3	0/5	/	0/5	/	0/10	/	7/14		yes
	F1	*S09*	*S05*	3	/	0/5	/	0/10	/	4/20	11/15		yes
	F1	*S09*	*S04*	2	/	/	0/10	/	/	1/15	8/10		yes
	F1	*S09*	*S01*	1	/	0/10	/	/	/	/	5/5		yes
	F1	*S09*	*S13*	2	/	/	0/14	0/10	/	/	2/5		yes
				Controls	67/85	11/15	12/14	5/10	5/5	4/4			
				S-shared *vs.* Controls									
				Expressed in pollen ?	yes	yes	yes	yes	yes	yes			

Figure S9 Summary of cross-pollinations realized for individuals from collection F0 and F1 having *S*-haplotype *S09* (*A06-A07*). See Figure S1 for legend details.
