# Supplementary material for: Self-Incompatibility in Brassicaceae: Identification and Characterization of SRK-Like Sequences Linked to the S-Locus in the Tribe Biscutelleae
Source: G3 (Bethesda). 2013 Dec 23;4(6):983–92. doi: 10.1534/g3.114.010843 (PMC4065267; doi:10.1534/g3.114.010843)
Supplement: Supporting Information [file supp_4.6.983_FigureS10.pdf]

S10

				Pollen donors F0		Pollen donors F1				
	S-haplotypes		1	*S10*	*S10*	*S10*	*S10*	Controls	S-shared vs. Controls	Expressed in stigma ?
			2	*S05*	*S08*	*S02*	*S01*			
	1	2	Plants	2	1	2	3			
Pollen receptors (stigma) F0	*S10*	*S05*	2	0/20	0/5* 5/5**	/	/	113/140		yes*/no**
Pollen receptors (stigma) F0	*S10*	*S08*	1	9/10**	0/5	/	/	78/90		?**
Pollen receptors (stigma) F1	*S10*	*S02*	2	/	/	/	1/20	23/30		yes
Pollen receptors (stigma) F1	*S10*	*S01*	3	/	/	14/25**	/	1/5		?**
	Controls			126/150	77/95	15/18	3/5			
	S-shared *vs.* Controls									
	Expressed in pollen ?			?**	yes*/ no**	?**	yes			

Figure S10 Summary of cross-pollinations realized for individuals from collection F0 and F1 having *S*-haplotype *S10* (*B05*). See Figure S1 for legend details.
