# Supplementary material for: Self-Incompatibility in Brassicaceae: Identification and Characterization of SRK-Like Sequences Linked to the S-Locus in the Tribe Biscutelleae
Source: G3 (Bethesda). 2013 Dec 23;4(6):983–92. doi: 10.1534/g3.114.010843 (PMC4065267; doi:10.1534/g3.114.010843)
Supplement: Supporting Information [file supp_4.6.983_FigureS11.pdf]

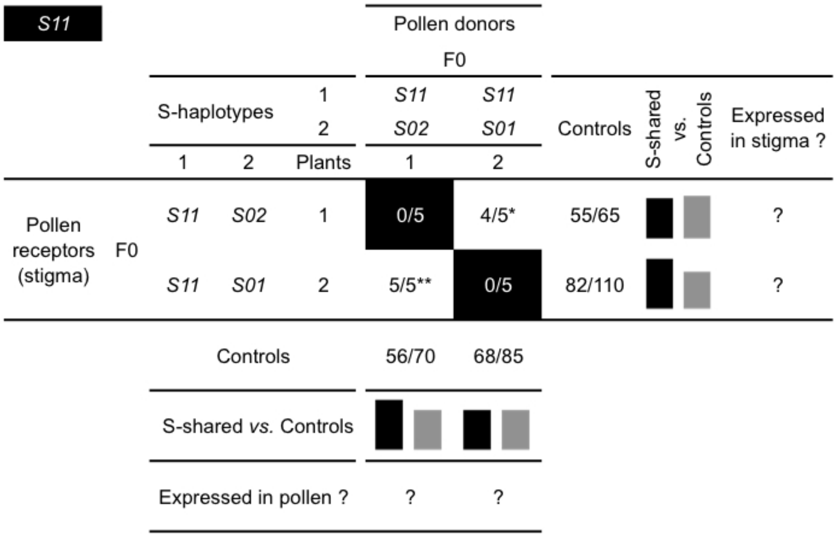

Figure S11 Summary of cross-pollinations realized for individuals from collection F0 and F1 having *S*-haplotype *S11* (*B09-B12*). See Figure S1 for legend details.
