# Supplementary material for: Self-Incompatibility in Brassicaceae: Identification and Characterization of SRK-Like Sequences Linked to the S-Locus in the Tribe Biscutelleae
Source: G3 (Bethesda). 2013 Dec 23;4(6):983–92. doi: 10.1534/g3.114.010843 (PMC4065267; doi:10.1534/g3.114.010843)
Supplement: Supporting Information [file supp_4.6.983_FigureS12.pdf]

S12

				Pollen donors F1					
		S-haplotypes 1		*S12*	*S12*	*S12*			
		S-haplotypes 2		*S07*	*S02*	*S01*	Controls	S-shared vs. Controls	Expressed in stigma ?
		1	2	Plants: 1	4	1			
		**1**	**2**	**Plants**					
Pollen receptors (stigma)	F1	*S12*	*S07*	1		5/5		NA	NA	?
		S12	*S02*	4	5/5		0/5*	7/10		yes*
		S12	*S01*	1		5/5		4/5		?

	S12 S07	S12 S02	S12 S01
Controls	NA	10/10	3/4
S-shared *vs.* Controls	NA		
Expressed in pollen ?	?	?	yes*

Figure S12 Summary of cross-pollinations realized for individuals from collection F0 and F1 having *S*-haplotype *S12* (*B17*-*B18*). See Figure S1 for legend details.

Haplotype *S12* was not typed in collection F1 because of no available specific primers for *B17* or *B18*. The presence of *S12* in collection F1 was thus deduced from the segregation pattern in offspring of individual 9 of (genotype *S03*-*S12;* see Figure 3 and Table 3). In collection F1, we thus considered that *S12* was present when *S03* (*B06*) was absent.
