# Supplementary material for: Self-Incompatibility in Brassicaceae: Identification and Characterization of SRK-Like Sequences Linked to the S-Locus in the Tribe Biscutelleae
Source: G3 (Bethesda). 2013 Dec 23;4(6):983–92. doi: 10.1534/g3.114.010843 (PMC4065267; doi:10.1534/g3.114.010843)
Supplement: Supporting Information [file supp_4.6.983_FigureS13.pdf]

S13

					Pollen donors F1				
		S-haplotypes		1	*S13*	*S13*	Controls	S-shared vs. Controls	Expressed in stigma ?
				2	*S09*	*S03*			
		1	2	Plants	2	2			
Pollen receptors (stigma)	F1	*S13*	*S09*	2	/	0/20	2/5		yes
		S13	*S03*	2	0/20	/	9/9		yes
		Controls			4/4	8/10			
		S-shared *vs.* Controls							
		Expressed in pollen ?			yes	yes			

Figure S13 Summary of cross-pollinations realized for individuals from collection F0 and F1 having *S*-haplotype *S13* (*A04*).
