# Supplementary material for: Self-Incompatibility in Brassicaceae: Identification and Characterization of SRK-Like Sequences Linked to the S-Locus in the Tribe Biscutelleae
Source: G3 (Bethesda). 2013 Dec 23;4(6):983–92. doi: 10.1534/g3.114.010843 (PMC4065267; doi:10.1534/g3.114.010843)
Supplement: Supporting Information [file supp_4.6.983_FigureS14.pdf]

S02 vs. S07

Pollen receptors (stigma)		S-haplotypes 1	S-haplotypes 2	Plants	Pollen donnors F0	F1										
				S-haplotype 1	**S02**	**S07**	**S07**	**S07**	**S07**	**S02**	**S02**	**S02**	**S02**	**S02**	**S02**	**S02**
				S-haplotype 2	**S07**	S04**	S03*	S01	S12***	S03*	S01	S09	S04**	S08	S10	S12
		1	2	Plants	1	2	2	2	1	5	4	3	3	2	2	4
	F0	**S02**	**S07**	1	/	/	/	0/5	/	/	/	0/5	0/5	/	/	/
	F1	**S07**	S04**	2	/	/	1/20	/	/	1/5	8/19	/	1/10**	/	/	/
	F1	**S07**	S03*	2	0/5	0/19	/	/	/	0/5*	/	/	5/5	/	7/8	5/5
	F1	**S07**	S01	2	0/5	/	/	/	8/18***	4/5	/	/	/	/	/	/
	F1	**S07**	S12	1	/	/	/	0/9	/	/	/	/	/	/	/	5/5
	F1	**S02**	S03*	5	/	3/5	0/5*	8/10	/	/	/	0/48	0/19	/	/	1/55
	F1	**S02**	S01	4	/	8/15	/	/	/	/	/	/	1/39	/	/	/
	F1	**S02**	S09	3	0/5	/	/	/	/	0/60	/	/	/	/	/	/
	F1	**S02**	S04**	3	0/5	0/10**	5/5	/	/	0/20	1/44	/	/	/	/	/
	F1	**S02**	S08	2	/	/	/	/	/	/	/	/	/	/	0/20	/
	F1	**S02**	S10	2	/	/	8/10	/	/	/	/	/	/	0/20	/	/
	F1	**S02**	S12	4	/	/	2/5	/	5/5	0/78	/	/	/	/	/	/

Figure S14 Cross-pollinations between plants having haplotype *S02* and plants having haplotype *S07* are mostly successful, indicating that *S02* and *S07* are distinct functional *S*-haplotypes, while sharing sequence *A01* in common. See Figure S1 for legend details.
